# Supplementary material for: How do gender norms contribute to stunting in Ntchisi District, Malawi? a qualitative study
Source: PLoS One. 2024 Oct 22;19(10):e0290199. doi: 10.1371/journal.pone.0290199 (PMC11495627; doi:10.1371/journal.pone.0290199)
Supplement: Attachment — Submitted filename: REVIEWERS COMMENTS 4 PONE-D-23-21165.docx [file pone.0290199.s001.docx]

**REVIEWERS COMMENTS**

Generally, the study titled “How do gender norms contribute to stunting in Ntchisi District, Malawi? A qualitative study” was well-researched, very relevant, scientific, and very informative, it raised pertinent issues regarding the impact of gender norms on stunting in children.

The authors demonstrated excellent mastery of the study. However, few minor corrections are needed before the manuscript proceeds for publication.

Please find below the area that needs clarification and improvement.

In the abstract, the study appears not to have target population.

Lines 48& 49	Defined by the World Health Organization (WHO) as “deficiencies, excesses or imbalances in a person’s intake of energy and/or nutrients”- What was defined by WHO? The statement needs to be reconstructed.

Line 112 While reduced from 47% in 2010,(29) the prevalence of stunting in children under-five years in………………..

Line 113 . Among one of the most poorly…………………

Lines 181-183 Ethics approval was granted by the Trinity Health Policy and Management, Centre for Global Health (HP&M/CGH) Research Ethics Committee (REC) and by the Ntchisi District Council Secretariat, including the District Health Office (DHO) and the District Agriculture Office (DAO). Informed verbal……………

Line 199 a mean age of 26 while fathers had a mean age of 38. Participants reported………………

Lines 200 & 201 Most mothers and fathers were educated to primary level only, with some to second level.

Line 332 Discussion and Conclusion

# Lines 439 till end References

***Summary:*** The topic and the discuss appears not to tally, there are no information on feeding pattern of the children and their nutritional status. Secondly, is the study a purposive sampling of parents with stunted children? Thirdly, there is no correlational study to show the impact of gender norm on children’s nutritional status, so the study appears to be inconclusive.
