# Supplementary material for: How do gender norms contribute to stunting in Ntchisi District, Malawi? a qualitative study
Source: PLoS One. 2024 Oct 22;19(10):e0290199. doi: 10.1371/journal.pone.0290199 (PMC11495627; doi:10.1371/journal.pone.0290199)
Supplement: Attachment — Submitted filename: PONE-D-23-21165.docx [file pone.0290199.s002.docx]

PLOS ONE

**How do gender norms contribute to stunting in Ntchisi District, Malawi? A qualitative study**

--Manuscript Draft--

| **Manuscript Number:** | PONE-D-23-21165 |
| --- | --- |
| **Article Type:** | Research Article |
| **Full Title:** | How do gender norms contribute to stunting in Ntchisi District, Malawi? A qualitative study |
| **Short Title:** | Gender norms and stunting in Ntchisi, Malawi. |
| **Corresponding Author:** | Frédérique Vallières, Ph.D. Trinity College Dublin, Ireland Dublin, IRELAND |
| **Keywords:** | Stunting; gender norms; malnutrition; Malawi; children; UNICEF Conceptual Framework for Malnutrition |
| **Abstract:** | Background and Aim  Despite adequate food production and nutrition intervention coverage, stunting remains an enduring problem in Ntchisi, Malawi. Globally, gender and social norms are known to influence nutritional outcomes in children. This study explores how gender norms contribute to child stunting, in Ntchisi district, Central Malawi.  Research Methods  Informed by the UNICEF Framework for Malnutrition, nine focus group discussions were conducted with mothers (n=24), fathers (n=23), and members of policy and health treatment committees (n=21), spanning three different areas of Ntchisi district. Data were analysed through inductive thematic analysis, guided by the framework for Research in Gender and Ethics (RinGs).  Results  Three primary themes were identified: 1) gender unequal decision making on the consumption, sale and distribution of food; 2) enshrined community norms influence feeding practices underpinned by gender-based violence; and 3) policy disconnections and gaps that reinforce gender norms regarding nutrition. Themes encompassed practices across household, health treatment, and policy level.  Conclusion  Gender norms that underpin inequalities in decision making for production and consumption of food undermine children’s nourishment and contributes towards sustained child malnutrition in Ntchisi. Existing policy documents should revise their guidelines to incorporate gender norms as key determinants of malnutrition. |
| **Order of Authors:** | Whitney Mphangwe, MSc |
|  | Ann Nolan, PhD |
|  | Frédérique Vallières, PhD |
|  | Mairéad Finn, PhD |
| **Additional Information:** |  |
| **Question** | **Response** |
| **Financial Disclosure**  Enter a financial disclosure statement that describes the sources of funding for the | The author(s) received no specific funding for this work. |

| work included in this submission. Review the [submission guidelines](http://journals.plos.org/plosone/s/submission-guidelines#loc-financial-disclosure-statement) for detailed requirements. View published research articles from [*PLOS ONE*](http://journals.plos.org/plosone/) for specific examples.  This statement is required for submission and **will appear in the published article** if the submission is accepted. Please make sure it is accurate. |  |
| --- | --- |
| **Unfunded studies**  Enter: *The author(s) received no specific funding for this work.*  **Funded studies**  Enter a statement with the following details:   - Initials of the authors who received each award - Grant numbers awarded to each author - The full name of each funder - URL of each funder website - Did the sponsors or funders play any role in the study design, data collection and analysis, decision to publish, or preparation of the manuscript? - **NO** - Include this sentence at the end of your statement: *The funders had no role in study design, data collection and analysis, decision to publish, or preparation of the manuscript.* - **YES** - Specify the role(s) played. |  |
| * typeset |  |
| **Competing Interests**  Use the instructions below to enter a competing interest statement for this submission. On behalf of all authors, disclose any [competing interests](http://journals.plos.org/plosone/s/competing-interests) that could be perceived to bias this work—acknowledging all financial support and any other relevant financial or non- financial competing interests.  This statement is required for submission and **will appear in the published article** if the submission is accepted. Please make sure it is accurate and that any funding sources listed in your Funding Information | The authors have declared that no competing interests exist. |

| later in the submission form are also declared in your Financial Disclosure statement.  View published research articles from  [*PLOS ONE*](http://journals.plos.org/plosone/) for specific examples. |  |
| --- | --- |
| **NO authors have competing interests**  Enter: *The authors have declared that no competing interests exist.*  **Authors with competing interests**  Enter competing interest details beginning with this statement:  *I have read the journal's policy and the authors of this manuscript have the following competing interests: [insert competing interests here]* |  |
| * typeset |  |
| **Ethics Statement**  Enter an ethics statement for this submission. This statement is required if the study involved:   - Human participants - Human specimens or tissue - Vertebrate animals or cephalopods - Vertebrate embryos or tissues - Field research   Write "N/A" if the submission does not require an ethics statement.  General guidance is provided below. Consult the [submission guidelines](http://journals.plos.org/plosone/s/submission-guidelines#loc-guidelines-for-specific-study-types) for detailed instructions. **Make sure that all information entered here is included in the Methods section of the manuscript.** | Ethics approval was granted by the Trinity Health Policy and Management, Centre for Global Health (HP&M/CGH) Research Ethics Committee (REC) and by the Ntchisi District Council Secretariat, including the District Health Office (DHO) and the District Agriculture Office (DAO). Informed consent was sought from participants, who were fully briefed about the study aims and objectives, the voluntary nature of their participation, and that they could opt out at any time without penalty. |

| **Format for specific study types**  **Human Subject Research (involving human participants and/or tissue)**   - Give the name of the institutional review board or ethics committee that approved the study - Include the approval number and/or a statement indicating approval of this research - Indicate the form of consent obtained (written/oral) or the reason that consent was not obtained (e.g. the data were analyzed anonymously)   **Animal Research (involving vertebrate animals, embryos or tissues)**   - Provide the name of the Institutional Animal Care and Use Committee (IACUC) or other relevant ethics board that reviewed the study protocol, and indicate whether they approved this research or granted a formal waiver of ethical approval - Include an approval number if one was obtained - If the study involved *non-human primates*, add *additional details* about animal welfare and steps taken to ameliorate suffering - If anesthesia, euthanasia, or any kind of animal sacrifice is part of the study, include briefly which substances and/or methods were applied   **Field Research**  Include the following details if this study involves the collection of plant, animal, or other materials from a natural setting:   - Field permit number - Name of the institution or relevant body that granted permission |  |
| --- | --- |
|  |  |
| **Data Availability**  Authors are required to make all data underlying the findings described fully available, without restriction, and from the time of publication. PLOS allows rare exceptions to address legal and ethical concerns. See the [PLOS Data Policy](http://journals.plos.org/plosone/s/data-availability) and [FAQ](http://journals.plos.org/plosone/s/data-availability#loc-faqs-for-data-policy) for detailed information. | No - some restrictions will apply |

| A Data Availability Statement describing where the data can be found is required at submission. Your answers to this question constitute the Data Availability Statement and **will be published in the article**, if accepted. |  |
| --- | --- |
| **Important:** Stating ‘data available on request from the author’ is not sufficient. If your data are only available upon request, select ‘No’ for the first question and explain your exceptional situation in the text box. |  |
| Do the authors confirm that all data underlying the findings described in their manuscript are fully available without restriction? |  |
| **Describe where the data may be found in full sentences. If you are copying our sample text, replace any instances of XXX with the appropriate details.**   - If the data are **held or will be held in a public repository**, include URLs, accession numbers or DOIs. If this information will only be available after acceptance, indicate this by ticking the box below. For example: *All XXX files are available from the XXX database (accession number(s) XXX, XXX.*). - If the data are all contained **within the manuscript and/or Supporting Information files**, enter the following: *All relevant data are within the manuscript and its Supporting Information files.* - If neither of these applies but you are able to provide **details of access elsewhere**, with or without limitations, please do so. For example:   *Data cannot be shared publicly because of [XXX]. Data are available from the XXX Institutional Data Access / Ethics Committee (contact via XXX) for researchers who meet the criteria for access to confidential data.*  *The data underlying the results presented in the study are available from (include the name of the third party* | The pseudo-anonymised qualitative data is available from the first author, upon reasonable request. |

| *and contact information or URL).*   - This text is appropriate if the data are owned by a third party and authors do not have permission to share the data.   * typeset |  |
| --- | --- |
| Additional data availability information: |  |

Manuscript	[Click here to access/download;Manuscript;29.08.23 Manuscript_Stunting_UNICEF_Gender_Malawi (1).docx](https://www.editorialmanager.com/pone/download.aspx?id=34048664&guid=fadbadcd-efb6-4f4a-8b5b-fdd37bbe71a0&scheme=1)

# 1 How do gender norms contribute to stunting in Ntchisi District, Malawi? A qualitative study

2 Whitney Mphangwe^1,2^, Ann Nolan^1^, Frédérique Vallieres^3,*^, Mairéad Finn^3,4^
3 [mphangww@tcd.ie](mailto:mphangww@tcd.ie); [finnm2@tcd.ie](mailto:finnm2@tcd.ie)
4 ^1^ Trinity Centre for Global Health, School of Medicine, Trinity College Dublin
5 ^2^ Food and Nutrition Officer, Ministry of Agriculture, Irrigation and Water Development
6 ^3^ Trinity Centre for Global Health, School of Psychology, Trinity College Dublin.
7 ^4^ Department of Public Health and Epidemiology, Royal College of Surgeons in Ireland
8 *Corresponding Author 9

10

11 Whitney Mphangwe
12 Ministry of Agriculture, Irrigation and Water Development
13 Ntchisi District Agriculture Office
14 P.O. Box 37
15 Ntchisi
16 Malawi 17
18 Mairéad Finn
19 Trinity Centre for Global Health, University of Dublin, Trinity College
20 7 – 9 Leinster Street South
21 Dublin 2
22 Ireland 23

# 24 How do gender norms contribute to stunting in Ntchisi District, Malawi? A qualitative study

25 **Abstract**
26 **Background and Aim:** Despite adequate food production and nutrition intervention coverage,
27 stunting remains an enduring problem in Ntchisi, Malawi. Globally, gender and social norms are known
28 to influence nutritional outcomes in children. This study explores how gender norms contribute to
29 child stunting, in Ntchisi district, Central Malawi.
30 **Research Methods:** Informed by the UNICEF Framework for Malnutrition, nine focus group
31 discussions were conducted with mothers (n=24), fathers (n=23), and members of policy and health
32 treatment committees (n=21), spanning three different areas of Ntchisi district. Data were analysed
33 through inductive thematic analysis, guided by the framework for Research in Gender and Ethics
34 (RinGs).
35 **Results:** Three primary themes were identified: 1) gender unequal decision making on the
36 consumption, sale and distribution of food; 2) enshrined community norms influence feeding practices
37 underpinned by gender-based violence; and 3) policy disconnections and gaps that reinforce gender
38 norms regarding nutrition. Themes encompassed practices across household, health treatment, and
39 policy level.
40 **Conclusion:** Gender norms that underpin inequalities in decision making for production and
41 consumption of food undermine children’s nourishment and contributes towards sustained child
42 malnutrition in Ntchisi. Existing policy documents should revise their guidelines to incorporate gender
43 norms as key determinants of malnutrition.
44 **Keywords:**	Stunting, Gender Norms, Malnutrition, Malawi, Children, UNICEF Conceptual
45 Framework for Malnutrition

# 46 How do gender norms contribute to stunting in Ntchisi District, Malawi? A qualitative study

47 **Introduction**
48 Defined by the World Health Organization (WHO) as “deficiencies, excesses or imbalances in a
49 person’s intake of energy and/or nutrients”(1) the concept of malnutrition incorporates under-
50 nutrition, micro-nutrient related malnutrition, and over-nutrition. Malnutrition remains a topic of
51 global concern, with 20% of the global under-five population (149.2 million) currently ‘stunted,’ that
52 is, a low height-for-age index,(2) and where malnutrition accounts for 53% of under-5 mortality.(3)
53 Stunting causes long-term impairment including poor school performance in childhood, reduced
54 productivity later in life, and an increased risk of chronic non-communicable illnesses; and, in the
55 worst cases, death.(4)
56 Numerous international policy commitments seek to address malnutrition. The United Nations
57 (UN) Sustainable Development Goals (SDGs), particularly SDG2, aspire to improve agriculture and food
58 systems to feed and nourish malnourished populations.(5) Likewise, the WHO’s 2018 Global Nutrition
59 Target (GNT) sought to reduce the number of stunted children by 40%(6) and the UN’s 2015 General
60 Assembly declared 2015-2025 to be the decade of nutrition, a commitment by member states to
61 implement policies, programs, and investments to eliminate malnutrition in all its forms. Scaling Up
62 Nutrition (SUN) – a global movement working with the UN, civil society, business and donor networks
63 to tackle malnutrition - promotes exclusive care and investment in child care globally, particularly in
64 the first 1000 days of life.(7)
65 Established determinants of malnutrition include poor maternal nutrition and young child
66 feeding practices;(8, 9) young mothers’ age at first birth, which is linked to higher parity and poorer
67 birth spacing practices;(10) depleted nutrition for mothers experiencing successive pregnancies and
68 lactation,(8) associated with poorer feeding practices;(11) subclinical infections from exposures to
69 contaminated environments;(12)^,(13)^ and infectious diseases such as malaria, which can compound
70 short term loss of weight and contribute to long term stunting.(14) Malnutrition is thus determined
71 by the complex interaction of multiple social, economic, and cultural factors. These factors are
72 reflected in UNICEF’s Conceptual Framework of Maternal and Child Nutrition (Figure 1),(15) often
73 considered the primary global policy instrument to address the causes and consequences of
74 malnutrition and reduce its prevalence.(16) According to this framework, *basic-level* causes operate
75 in the broader context and include socio-economic and cultural context, human capital, and access to
76 potential resources; *underlying* causes operate at household and community level, including care and
77 feeding practices; and *immediate* causes relate to direct diet intake and the presence of disease.(16)
78 Recently updated in 2020, the framework now also recognizes contributors to good nutrition as being
79 either immediate, underlying, or enabling determinants.(17)

# 80 Figure 1: UNICEF Framework for Malnutrition

81 [INSERT FIGURE ONE HERE]

82

83 Gender refers to the socially constructed roles, behaviors, activities, and attributes that a given
84 society considers appropriate for men and women.(18) More recently, growing attention has been
85 brought to the impact of gender norms on child malnutrition and stunting.(19) In many settings,
86 women still cannot exercise freedom and autonomy of choice, including within their own
87 households,(14) with the extent to which a mother can make independent decisions negatively
88 associated with the likelihood of childhood stunting.(19) Similarly, granting women greater decision-
89 making power is associated with notable improvements in their children’s height-for-age index.(20)
90 Likewise, analysis of gender relations in norms and decision-making has recently come to the fore in
91 explaining the endurance of problematic health outcomes.(21) Indeed, positive social and cultural
92 norms (which include gender) are recognized as one of the most enabling determinants of improved
93 nutrition for women and children.(17)
94 In recognition of the role of gender and social norms in determining nutritional outcomes in
95 children, several frameworks have been created to guide gender analyses within health and nutritional
96 programming,(22-24) which differ in their focus on types of power or area of the health system.
97 Specifically, the Research in Gender and Ethics (RinGS) Framework articulates gender differences as
98 unfolding across four domains: access to resources; division of labour, roles, and everyday practices;
99 social norms and values; and rules and decision making.(25, 26) It is considered comprehensive for
100 examining social and structural elements of gender interacting with health.(21)

# 101 Malnutrition in Malawi

102 Categorized as one of the poorest countries in the world by the World Bank,(27) Malawi is a Southern
103 African country home to over 18 million inhabitants. A signatory to the SUN movement since 2011,
104 the Malawian Government applies UNICEF’s Conceptual Framework for Malnutrition (1990) to
105 address malnutrition.(16) Moreover, the Malawi Multi-Sectoral National Nutrition Policy (MSNNP)
106 sets out the country’s priority areas for implementing nutrition interventions across the country.(28)
107 Specifically, these priority areas include (i) the prevention of under-nutrition, (ii) achieving gender
108 equality, equity protection, participation and empowerment, and (iii) the treatment and control of
109 malnutrition.(28) The MSNNP outlines best practices to end malnutrition, detailing policy and
110 implementation plans to address causes of malnutrition at household, health treatment, and policy
111 levels.(28)
112 While reduced from 47% in 2010,(29) the prevalence of stunting in children under-five years in
113 Malawi remains a worrying 37% in 2015, the most recent available data. Among one of the most poorly
114 affected districts in the country is Ntchisi, where 40% of the population are malnourished.(29)
115 Paradoxically, Ntchisi is also considered one of the most household food secure districts in the
116 country.(30, 31) Being food secure defined as people having at all times physical, social and economic
117 access to food, safely consumed in sufficient quantity and quality for dietary needs and food
118 preferences, supported by an environment of adequate water and sanitation, health services and
119 care.(32, 33) Furthermore, exclusive breastfeeding rates in Ntchisi district for infants under six months
120 (61%) exceed the global average (40%).(29)^,^(2)
121 Evidence suggests that gender norms may play a role in Ntchisi’s observed malnutrition rates.
122 For example, in 2016, 47% of women were married before 18 years and 29% of adolescent girls were
123 mothers.(29, 34) Women were also found to be five times less likely to make independent decisions
124 when compared with men, a difference linked to variation in earning power.(35) There is evidence
125 that differences in gender norms have a detrimental impact on women’s health.(36) *How* differences
126 in gender norms may contribute to malnourishment and stunting in children, in Ntchisi, Malawi,
127 however, remain unexplored. This study therefore aimed to explore how gender norms play a role in
128 continued stunting in Ntchisi District, Malawi, and to contribute to a revised version of the UNICEF
129 Framework for Malnutrition to better reflect gender norms.
130 130

# 131 Materials and Methods

132 *Design and Sampling*
133 We employed a qualitative design, as part of a wider examination of the causes of observed stunting
134 in Ntchisi district. In February and March 2019, we conducted nine focus group discussions (FGDs) to
135 explore the role of gender norms in persistent child stunting across three different communities. FGDs
136 were selected as they are considered valuable for generating data on group social norms, while also
137 prioritising participants’ perspectives.(37) Each community was purposely selected to achieve
138 geographical spread. Six FGDs - two in each community - were conducted with mothers and fathers
139 as household members, one FGD was conducted with participants delivering nutrition health
140 treatment and two were conducted with participants working on nutrition policy. Therefore, nine
141 focus groups explored how gender norms might impact (mal)nutrition, reflecting household, health
142 treatment and nutrition policy spheres as articulated in the Malawi MSNNP.(28)
143 In total, 68 participants were recruited, with 6-9 people represented within each FGD.
144 Recruitment took place between 28^th^ of February and 9^th^ of March, 2019. The six FGDs with household
145 members comprised three with males (n=24) and three with females (n=23). Females were within the
146 reproductive age group, had at least one child under the age of five in the household, and were
147 recruited through Growth Monitoring Sessions. There was no age limitation for males, who were also
148 required to have at least one child under the age of five in the household. Men were recruited from
149 nearby social gathering venues, mostly at church choir gatherings and market points via word-of-
150 mouth by the government extension workers.
151 At health treatment level, participants (n=10) included five females and five males of the
152 Nutrition Rehabilitation Unit (NRU) Management Committee. This committee comprises nutritionist,
153 nurse, and home-help workers providing treatment to severe cases of malnutrition admitted to the
154 District Health Office. Policy level participants (n=10) also comprised five females and five males of
155 the District Agriculture Coordinating Committee (DAECC), a committee that oversees and coordinates
156 all agricultural interventions at the district level, and the District Nutrition Coordinating Committee
157 (DNCC), a committee responsible for coordinating all nutrition interventions at the district.
158 158
159 Data Collection Instruments
160 Semi-structured focus group discussion guides were developed with reference to the domains of the
161 UNICEF Framework of Malnutrition(16, 17), incorporating probes to ensure maximum investigation of
162 persistent stunting. The guides included general questions about daily activities, food and income;
163 resources and decision-making; and understanding of and perspectives on stunting. Discussions were
164 conducted by the first author in Chichewa, the local language. Two field assistants, one from the health
165 sector and the other from the agriculture sector, took notes of the participants’ answers throughout
166 the discussions as a backup for the audio recordings.
167 167
168 Data Management and Analysis
169 FGDs were recorded, transcribed verbatim,(38) and translated from Chichewa to English with close
170 reference to audio files and notes. All personal identifiers were removed with all transcripts
171 anonymized. Following a thorough review of the transcripts for familiarity, analysis comprised three
172 phases of open (i.e. inductive) coding, categorization, and abstraction. Transcripts were coded
173 according to the steps of Braun and Clarke(39), with common phrases assigned codes, then inductively
174 organized into categories and themes. A framework for gender analysis,(25, 26) informed by Research
175 in Gender and Ethics (RinGs)(22) guided analytic insights. Interpretation of the data was enhanced by
176 discussions between the first author, the field workers, and the last author to refine analytical
177 development and verify the conceptual coherence of the codes. All authors reviewed the categories
178 and themes.
179 179
180 Ethics, Validity and Reliability
181 Ethics approval was granted by the Trinity Health Policy and Management, Centre for Global Health
182 (HP&M/CGH) Research Ethics Committee (REC) and by the Ntchisi District Council Secretariat,
183 including the District Health Office (DHO) and the District Agriculture Office (DAO). Informed verbal
184 consent was sought from participants, who were fully briefed about the study aims and objectives.
185 Verbal consent was favoured to avoid embarrassment for those who could not write, as literacy levels
186 could not be established at the start of the interviews. Verbal consent was witnessed by the group
187 and field workers. At all times, participants were assured of the voluntary nature of their participation,
188 and that they could withdraw at any point in time without penalty.
189 The validity and reliability of the data was ensured through transcription, clear and transparent
190 coding, reflexivity, and cross-checking of themes between the lead author, the last author, and two
191 field assistants.(40) The second and third author validated the coherence of the thematic categories.
192 The first author, who carried out the data collection, is a nutritionist in Ntchisi District, and reflexive
193 attention was paid to the influence of her positionality throughout the data collection and analysis
194 phases.
195 195
196 196

# 197 Results:

198 **Participant Characteristics**
199 Participant mothers had a mean age of 26 while fathers had a mean age of 38. Participants reported
200 having five children, on average. Most mothers and fathers were educated to primary level only, with
201 some to second level. Agriculture was their main source of food and income. Among committee
202 members, half were employed by government while the other half were employed by non-
203 government organizations.

# 204 Exploring the Role of Gender Norms in Contributing to Malnutrition

205 Three core themes were identified in the data to illuminate the role of gender norms in contributing
206 to malnutrition and stunting in Ntchisi. These were: (i) gender unequal decision making on the
207 consumption, sale and distribution of food; (ii) enshrined community norms influence feeding
208 practices, underpinned by gender based violence; (iii) policy disconnections and gaps that reinforce
209 unequal gender norms regarding nutrition. Themes are presented in Figure 2, aligned to the RinGS
210 Framework for Gender Analysis.(21, 22) 211

# 212 Figure 2: Gender Norms Contributing to Stunting in Ntchisi District, Malawi

213 [INSERT FIGURE TWO HERE]

# 214 Gender Unequal Decision Making Shapes the Household Production and Consumption of Food

215 Power differences between fathers and mothers affected decisions about how farm produce is
216 utilized. The policy level focus groups explained how practices in subsistence agriculture affect what
217 to produce, the quantity to produce, and where to sell produce, all of which affect how much remains
218 for families to eat. Women in the district were reported to have lower decision-making power about
219 food production by comparison to men:
220 “Men dominate…they control what is cultivated and what to do with the produce. You find that
221 a woman might know that legumes are nutritious and essential for child growth… [but] when
222 the husband wants to sell, the [wife] will not resist.” DAECC FGD.
223 Describing their social context, household participants told of the different roles that mothers and
224 fathers take and how this impacts children’s nutrition. The participants recounted that within the
225 household, fathers are considered providers while mothers are nurturers. When describing their daily
226 routine, mothers mentioned household chores like cooking, feeding, laundry, tidying the house, and
227 collecting firewood and water, the latter usually from a great distance. On the other hand, fathers
228 reported daily work of gardening, casual tasks, socializing, and sourcing income for the household.
229 Fathers and mothers emphasised a clear distinction of roles in feeding children:
230 “Men search for food…we stay at home taking care of the children… cooking for and feeding
231 them. When men bring food, we cook.” Mothers FGD
232 “We take care of children in the sense that we search for food so that woman can cook it for
233 the children.” Fathers FGD.
234 However, fathers were characterized by participants as not health or nutrition minded in relation to
235 expenditure. Almost all mothers’ groups described that men in the community use their income on
236 pleasures rather than on actual needs.
237 “Sometimes, you can work hard to produce more. However, when you harvest and sell, the man
238 will spend it on drinking and other women. You cannot question how they spend it. They forget
239 about their own children. You face it all because the children usually stay at home with a
240 mother”. Mothers FGD.
241 Similarly, fathers indicated that when they harvest, they have several responsibilities to address.
242 Nutrition might not usually be the priority.
243 “A poor person always has a handful of things to take care of. When you get money…you don’t
244 remember what nutrition or nutritious food is about” Fathers FGD.
245 Differences between mothers and fathers in decision making about utilization of farm produce also
246 manifested at the health center. The health committee FGD recounted how in-patient cases of
247 malnutrition at the health centre are presented mostly by mothers, who attend with their children
248 while fathers stay at home. Extended stays can cause worry to mothers about further disruption to
249 home life and the oversale of food:
250 “The mothers wait for children here…we give them health talks every morning. Fathers usually
251 stay at home…[but]…when the mothers stay for some days, they start complaining thinking that
252 their husbands will marry another woman or get a chance to sell out farm produce and other
253 household assets. They just become so eager to go, and it is evident in how they act.” NRU FGD.
254 Although mothers in Ntchisi were primarily responsible for feeding children, they lacked decision-
255 making power over the production and consumption of food. Fathers, who held decision making
256 power, were not oriented towards nutritional needs for children. Furthermore, inequality in decision
257 making about food for children intensifies when women are not present.
258 258

# 259 Enshrined Community Norms Influence Feeding Practices, Underpinned by Gender Based Violence

260 Household focus groups reported that inequity in household decision making around food was
261 preserved by traditional norms of gender-based violence (GBV) in Ntchisi district. A clear and strong
262 description by participants was that women cooperate with men’s decisions for fear of GBV, and do
263 not speak out about GBV in fear of shaming their husbands and losing respect.
264 “In this area, when a woman has a brilliant idea, men do not want to appear cheap, so they do
265 not agree, whatever it is about…as a woman you just coil up and keep quiet otherwise you will
266 be beaten” Mothers FGDs
267 The supremacy of this social norm was bolstered by the status it confers on men in the community.
268 “Men here say things, and that is final…maybe it could be one out of ten men that would be
269 under petticoat government. If you allow your wife to be key in making decisions, your friends
270 will despise you…even young children would look down on you.” Fathers FGD.
271 District committee members recounted that cases of GBV are more prevalent at times of the year
272 when produce is sold. Gaps in power between mothers and fathers extended to a firm inequality in
273 autonomy, which impacted on children’s nutrition.
274 “Men are just bad. During the cultivating period, they work well with their wives, but when it is
275 selling time, they prefer to work alone and spend the money on drinking or other women…this
276 initiates conflicts that usually ends in abuse. Children are usually left with mothers that do not
277 have food supplies or money.” DNCC FGD.
278 Mothers and fathers highlighted that the clusters of friends and neighbours they lived among were
279 their primary sources of knowledge, indicating the strong traditional norms shaped how children were
280 taken care of. Gender based violent practices were encased in traditional community norms.
281 “Everybody does that…that’s how we do things here. Our parents teach us what they did so that
282 we grew up. When your child is born, they take you aside and teach you many things	we
283 apply that so that our children grow up too” Fathers FGD.
284 Thus, community adherence to traditional practice creates a challenge to do things differently
285 when feeding children. Rigid social norms of women required to be deferential to men were held in
286 place by traditional expectations and the threat of gender-based violence. These norms retained an
287 enduring social power in the community and impacted practices and decision making around food for
288 children.
289 289

# 290 Policy Disconnections and Gaps that Structurally Reinforce Gender Norms Regarding Nutrition

291 A disconnection between production and nutrition interventions was articulated by policy level
292 participants, with agricultural produce sold by subsistence farmers rather than retained for their
293 families. Participants explained that while the agriculture committee incorporates nutrition
294 interventions to its activities in theory; no nutrition achievements could be attributed to this work.
295 “We try to incorporate nutrition in our planning, but all detailed plans and achievements are
296 with the DNCC because we mainly focus on agriculture … however, nutrition is for DNCC, so
297 consult them for specific achievements.” DAECC FGD.
298 The disconnection indicates a wider aspect of the gender gap because agriculture interventions -
299 usually organized by men in the district - are implemented separately from general nutrition
300 interventions, targeting women as primary caregivers for children.
301 Men’s participation in the implementation of nutrition interventions was noted as a gap by
302 household level participants. Mothers encouraged targeting fathers for inclusion, highlighting that if
303 men were aware of nutrition issues, they would be more supportive at home and more informed
304 about nutrition-related decisions.
305 “Men should be taught. They are stubborn and irresponsible because they do not know and
306 understand. They should hear for themselves. They should be included in care groups. Now care
307 groups are only full of women” Mothers FGD
308 Despite father’s lack of involvement in nutrition reflecting the socio-cultural norms in Ntchisi,
309 men in the fathers’ focus groups indicated their willingness to participate in the sessions and learn
310 more about nutrition.
311 “The extension workers always teach women. The next training, please include us…we will come
312 and are happy to learn.” Fathers FGD
313 Impacts of trainings were also affected by education levels, specifically literacy, of the
314 population. Low levels of education were reported as a general feature of the population in Ntchisi,
315 affecting practices towards childcare. Health treatment level participants highlighted how literacy
316 levels affect the uptake of trainings.
317 “You can have a hospital, offer training in all these things…however, without school, it is all in
318 vain. You need some [education] to understand the importance of it all.” NRU FGD.
319 The Government’s role was further emphasized in reducing teen pregnancies and achievements
320 in nutrition education. Teen mothers face challenges in adequately nourishing themselves and their
321 children. Pressure is exacerbated when women have their first child at a young age, because it leads
322 to larger family size. Care demands of young children cannot be met if the mother has many children,
323 closely spaced together.
324 “I think the issue is that people have many children. You see some have eight, others nine. They
325 cannot afford to monitor if all ate or not. You would not know that one missed food because
326 they are too many..[…]… the Government should do more in reducing teen pregnancies…and
327 should invest in keeping girls in school.” NRU FGD.
328 Low levels of education undermine the household’s ability to meet the nutrition needs of children,
329 affecting both choices in health and well-being and the priority given to well-being. These trends are
330 also an indication of a broader structural context with unequal gender norms impacting women and
331 children.

# 332 Discussion and Conclusion

333 Despite a context of food security, interventions on nutritious feeding, and the development of
334 policies to target the eradication of undernutrition, children’s inadequate consumption of food in
335 Ntchisi is impacted by gendered practices and power dynamics. Specifically, differing roles between
336 men and women shape use of farm produce and feeding practices, enduring traditional social norms
337 preserved via the threat of gender-based violence and structurally bolstered through policy
338 disconnections, are all experienced as gender-related factors that impact on the nutrition of children
339 in Ntchisi.
340 Similar to Alemayehu et al.(14), and Kamiya et al.(41), women interviewed in Ntchisi district also
341 had limited power in making decisions about use of food within their households. Adhering to men’s
342 decisions regarding food production is a strong tradition that results in households being unable to
343 consume the right varieties and quantities. Although subsistence farming is generally characterized by
344 the consumption of produce,(42) the narratives in this study highlighted how food produced for
345 subsistence is sold and distributed rather than consumed.	Subsistence farming also connotes
346 adherence to indigenous knowledge and traditions in production and consumption, pointing again to
347 the embedded nature of traditional practices.(43)
348 Power differences between women and men were also evident in that women were involved
349 in harvesting food, but excluded from decisions on the use of the harvest. Conflict related to decisions
350 on use of harvest produce reportedly resulted in increased gender-based violence or a rupture in the
351 marriage, as participants recounted risks of both increased violence and fathers sometimes
352 abandoning their families. This trend has been identified elsewhere, where women characterized
353 themselves as ‘harvest wives’ in cases where their husbands abandoned them when the harvest was
354 complete.(44) A salient phenomenon that impacts the use of food is women’s lack of inclusion in
355 decisions on how harvested food will be used for the family, with a heightened risk of gender based
356 violence or relationship breakdown at this point.
357 While the government has an established role in imparting knowledge on food preparation and
358 feeding, enshrined gender norms undermine how women can practice their learning, as they are
359 pressured to adhere to men’s decisions. Women’s translation of their nutrition knowledge into
360 practice is also challenged by the high number of GBV cases reported in the district, more prevalent
361 during harvest time, also contributing to the limited contribution of agricultural produce to
362 nourishment of children. Consistent with previous findings, children’s dietary intake in Ntchisi is also
363 reportedly further compromised by mothers’ young age and a large number of children, with
364 consequent reduced care practices.(45) In the context where women do not make independent
365 decisions, it can be assessed that the lack of independence extends to decisions on family size and
366 birth control methods.
367 Father’s lack of involvement within Ntchisi was also found to be a manifestation of the social
368 cultural norms in the district. Participants across the sample suggested inclusion of men in community
369 trainings on infant and young child feeding. In a social context where fathers want to be respected for
370 not being “under petticoat government”, the cultural change required to incorporate men into
371 nutrition interventions is evident. A more targeted approach to addressing men’s roles in nutrition,
372 that takes account of the bearing of gender norms, is required.
373 In support of the UNICEF Conceptual Framework of Malnutrition, the findings from this study
374 demonstrate that gender dynamics indeed shape malnutrition across all three causes of malnutrition
375 articulated in the framework - basic, underlying, and intermediate. Practices at the underlying levels
376 of household and community related to the production and consumption of food were highly unequal
377 for men and women. At the basic level of socio-economic and cultural context, capital, and access to
378 resources, powerful norms bolstering inequity between women and men were evident. Unequal
379 practices within these domains were highly gendered, intersecting to shape lack of access to and use
380 of resources for the nourishment of children. These findings and considerations are presented in
381 Figure Three as an expanded version of the UNICEF Conceptual Framework for Maternal and Child
382 Malnutrition.

# 383 Figure 3: Factors Contributing to Stunting adapted to the 1990 UNICEF Conceptual Framework of

384 **Malnutrition**
385 [INSERT FIGURE 3 HERE]
386 The current study adds to the existing literature by illustrating that in Ntchisi, gender norms
387 manifest across basic, underlying, and intermediate levels, and not just at the basic level as currently
388 articulated within the UNICEF conceptualization. Accordingly, gender should be articulated across the
389 entire UNICEF	framework, and	the	framework	might	benefit	from	applying	the	WHO’s
390 recommendations for incorporating intersectional gender analysis.(25, 26) In line with the domains
391 within RiNGs framework, domains of ‘access to resources’; ‘divisions of labour, roles and everyday
392 practices’; ‘social norms and values’; ‘rules and decision making’(26) should also be incorporated into
393 the UNICEF framework to illuminate how unequal gender norms and roles unfold to explain continued
394 problems of nutrition for children.
395 The lack of policy coordination between agriculture and nutrition could contribute to
396 explanations of the disconnection between food production, food consumption, and nutrition status
397 in Ntchisi. Strengthening policy linkages between these domains would align with the 2017 Global
398 Nutrition Report(46) statement that achieving good nutrition means practicing agriculture that feeds
399 and nourishes a nation state. The Food and Agriculture Organization emphasizes the importance of
400 integrating food production and nutrition interventions in parallel, and the findings presented here
401 provide insights for practical implementation.(47, 48) Integrating nutrition and agriculture is
402 emphasized by the theme of the 2016 World Food Day (WFD) commemorations; “our health is not
403 only what we eat, it is primarily what we grow”.(49) Gender sensitivity must traverse these
404 connections. Male involvement in these connections is imperative, in recognition of the fact that they
405 hold decision making power and asset ownership. Mkandawire & Hendricks (2015) note that while it
406 is important to target women and children in nutrition interventions, the exclusion of men ignores the
407 fact that women are limited in terms of decision making powers and asset ownership.(50)
408 Taken together, our findings demonstrate how (mal)nutrition is impacted by the socially
409 constructed roles, behaviours, activities and attributes that a given society considers appropriate for
410 men and women, which can manifest as unequal power relations shaping access to and use of
411 food.(26) The	Malawi	MSNNP	cites	gender	equality, equity	protection, participation	and
412 empowerment(28) as its second priority area. The findings can be translated into policy actions on
413 under-nutrition and stunting under the umbrella of the MSNNP. Specifically, it is recommended that
414 government and development partners continue multi-sectoral implementation but put special focus
415 on male involvement.
416 The Malawi MSNNP 2018-2022 emphasizes the need to involve all line ministries and
417 stakeholders in reducing child under-nutrition. In line with the policy objectives, this study
418 recommends that Government and other NGOs connect programmes across household, health
419 treatment and policy levels in Ntchisi; connect agriculture and nutrition polices; and incorporate a
420 gender lens. In addition, education must underpin all interventions, especially so for girls experiencing
421 teen pregnancy. The successful implementation of nutrition programmes relies on an educated and
422 literate population.

# 423 Limitations

424 The research had several limitations. Firstly, interviews were conducted in Chichewa, the vernacular
425 language in Ntchisi, and transcripts were translated to English by the first author who speaks Chichewa
426 and English fluently. This translation process, however, might have introduced some mis-explanations
427 of meanings of terms. Second, the first author is a nutrition officer who works in the area under study.
428 While every effort was made to account for the researcher role, it is challenging to fully eliminate a
429 researchers' influence on participant responses. In this case, the researcher’s gender or role as a
430 nutritionist may have influenced the discussions. Thirdly, data collection comprised FGDs with policy,
431 health management and household members in the community, excluding interviews within
432 individual households. Future research may want to explore how intra-household dynamics impact on
433 the nutritional status of women.
434 434

# 435 Acknowledgements

436 We would like to thank the participants and fieldworkers who gave their time to this study, and
437 without whom this research would not have been possible.
438 438

# 439 References

440 1.	World Health Organisation. Malnutrition Factsheet. Geneva: World Health Organisation; 441	2020.
442 2.	UNICEF, WHO, World Bank Group. Levels and Trends in Child Malnutrition: UNICEF / WHO /
443 World Bank Group Joint Child Malnutrition Estimates. Key Findings of the 2018 Edition. New York:
444 United Nations Children’s Fund (UNICEF); 2018.
445 3.	UNICEF. Nutrition, Malnutrition: Current Status and Progress. Geneva: United Nations
446 Children's Fund (UNICEF); 2018.
447 4.	Dewey KG, Begum K. Long-term consequences of stunting in early life. Maternal & Child 448	Nutrition. 2011;7(s3):5-18.
449 5.	Aheto JMK, Keegan TJ, Taylor BM, Diggle PJ. Childhood Malnutrition and Its Determinants
450 among Under-Five Children in Ghana. Paediatric and Perinatal Epidemiology. 2015;29(6):552-61.
451 6.	de Onis M, Dewey KG, Borghi E, Onyango AW, Blössner M, Daelmans B, et al. The World Health
452 Organization's global target for reducing childhood stunting by 2025: rationale and proposed actions.
453 Matern Child Nutr. 2013;9 Suppl 2(Suppl 2):6-26.
454 7.	Bloem M, Pee Sd. Scaling up nutrition: a framework for action. Food Nutr Bull. 2010;31(1):178- 455	86.

456	8.	Sullivan LM, Sakayan M, Cernak K. 1,000 Days: Mobilizing Investments for Healthier, More

457	Prosperous Futures. ZERO TO THREE. 2018;38(4):31-6.

458	9.	Kominiarek MA, Rajan P. Nutrition Recommendations in Pregnancy and Lactation. Med Clin 459	North Am. 2016;100(6):1199-215.

460 10.	Gupta A, Suri S, Dadhich J, Trejos M, Nalubanga B. The world breastfeeding trends initiative:
461 implementation of the global strategy for infant and young child feeding in 84 countries. Journal of
462 public health policy. 2019;40(1):35-65.
463 11.	Sankar MJ, Sinha B, Chowdhury R, Bhandari N, Taneja S, Martines J, et al. Optimal
464 breastfeeding practices and infant and child mortality: a systematic review and meta‐analysis. Acta 465	paediatrica. 2015;104:3-13.

466	12.	Cumming O, Cairncross S. Can water, sanitation and hygiene help eliminate stunting? Current

467	evidence and policy implications. Maternal & Child Nutrition. 2016;12(S1):91-105.

468	13.	Tickell KD, Pavlinac PB, John-Stewart GC, Denno DM, Richardson BA, Naulikha JM, et al. Impact

469	of Childhood Nutritional Status on Pathogen Prevalence and Severity of Acute Diarrhea. Am J Trop 470	Med Hyg. 2017;97(5):1337-44.

471	14.	Alemayehu M, Tinsae F, Haileslassie K, Seid O, Gebregziabher G, Yebyo H. Undernutrition

472	status and associated factors in under-5 children, in Tigray, Northern Ethiopia. Nutrition. 2015;31(7- 473	8):964-70.

474 15.	Reinhardt K, Fanzo J. Addressing Chronic Malnutrition through Multi-Sectoral, Sustainable
475 Approaches: A Review of the Causes and Consequences. Front Nutr. 2014;1:13.
476 16.	UNICEF. UNICEF Conceptual Framework for Malnutrition. 2008.
477 17.	World Health Organisation. UNICEF Conceptual Framework on Maternal and Child Nutrition.
478 New York: World Health Organisation; 2021.
479 18.	World Health Organisation. Gender [Internet]. Available from: : [https://www.who.int/health-](https://www.who.int/health-topics/gender)
480 [topics/gender;](https://www.who.int/health-topics/gender) 2022.
481 19.	Holmes AV, Chin NP, Kaczorowski J, Howard CR. A barrier to exclusive breastfeeding for WIC
482 enrollees: limited use of exclusive breastfeeding food package for mothers. Breastfeeding Medicine. 483	2009;4(1):25-30.
484 20.	Jesson J, Coulibaly A, Sylla M, N'Diaye C, Dicko F, Masson D, et al. Evaluation of a Nutritional
485 Support Intervention in Malnourished HIV-Infected Children in Bamako, Mali. JAIDS Journal of
486 Acquired Immune Deficiency Syndromes. 2017;76:14Y 157.
487 21.	Morgan R, Tetui M, Muhumuza Kananura R, Ekirapa-Kiracho E, George AS. Gender dynamics
488 affecting maternal health and health care access and use in Uganda. Health Policy and Planning. 489	2017;32(suppl_5):v13-v21.

490	22.	Morgan R, George A, Ssali S, Hawkins K, Molyneux S, Theobald S. How to do (or not to do)…

491	gender analysis in health systems research. Health Policy Plan. 2016;31(8):1069-78.

492	23.	Jhpiego. Gender Analysis Toolkit for Health Systems. Baltimore: Jhpiego; 2016.

493	24.	Warren H. Using gender-analysis frameworks: theoretical and practical reflections. Gender & 494	Development. 2007;15(2):187-98.

495	25.	World Health Organisation. Taking sex and gender into account in emerging infectious

496	diseases programmes: An analytical framework. Geneva: World Health Organisation; 2011.

497	26.	World Health Organisation. Incorporating Intersectional Gender Analysis into Research on

498	Infectious Diseases of Poverty: A Toolkit for Health Researchers. Geneva: World Health Organisation; 499	2020.

500 27.	World Bank. World Development Indicators. 2019.
501 28.	Government of Malawi. National Multi-Sector Nutrition Policy 2018 - 2022. Lilongwe:
502 Government of Malawi; 2018.
503 29.	Government of Malawi National Statistical Office. Malawi Demographic and Health Survey.
504 Zomba: Malawi National Statistical Office; 2017.
505 30.	Committee GoMVA. The Integrated Food Security Phase Classification (IPC) in Malawi:
506 Findings of the 2017 Assessment and Analysis. Lilongwe: Government of Malawi; 2017.
507 31.	World Food Programme. Household Food Security in Malawi: Results from a Comprehensive
508 Food Security Survey Malawi: World Food Programme; 2018.
509 32.	World	Bank.	What	is	Food	Security?	2022	[Available	from:
510 <https://www.worldbank.org/en/topic/agriculture/brief/food-security-update/what-is-food-security>.
511 33.	Dressel A, Mkandawire E, Gondwe KW, Mkandawire-Valhmu L, Nordin S, Vilakazi N, et al. The
512 intersection of food insecurity and health for rural Malawian women at the end of life. International
513 Journal of Palliative Nursing. 2020;26(7):372-82.
514 34.	Government of Malawi, Policy AIfD. Child Marriage in Malawi Factsheet. Lilongwe:
515 Government of Malawi Ministry of Gender, Children, Disability and Social Welfare; 2017.
516 35.	O’’Neil T, Kanyongolo N, Wales J, Walita Mkandawire M. Women and Power: Representation
517 and Influence in Malawi’s Parliament. London: Overseas Development Institute; 2016.
518 36.	Lodenstein E, Pedersen K, Botha K, Broerse JEW, Dieleman M. Gendered norms of
519 responsibility: reflections on accountability politics in maternal health care in Malawi. International
520 Journal for Equity in Health. 2018;17(1):131.
521 37.	Stewart DW, Shamdasani PN. Focus Groups: Theory and Practice. California: SAGE; 2015.
522 38.	Hennink M, Hutter I, Bailey A. Qualitative Research Methods. 2nd Edition ed. London: SAGE
523 Publications Ltd.; 2020.
524 39.	Braun V, Clarke V. Using thematic analysis in psychology. Qualitative Research in Psychology. 525	2006;3(2):77-101.

526	40.	Leung L. Validity, reliability, and generalizability in qualitative research. Journal of family

527	medicine and primary care. 2015;4(3):324-7.

528	41.	Kamiya Y, Nomura M, Ogino H, Yoshikawa K, Siengsounthone L, Xangsayarath P. Mothers’

529	autonomy and childhood stunting: evidence from semi-urban communities in Lao PDR. BMC Women's 530	Health. 2018;18(1):70.

531	42.	Sibhatu KT, Qaim M. Rural food security, subsistence agriculture, and seasonality. PloS one. 532	2017;12(10):e0186406.

533 43.	Rankoana SA, editor The Use of Indigenous Knowledge in Subsistence Farming: Implications
534 for Sustainable Agricultural Production in Dikgale Community in Limpopo Province, South Africa2017.
535 44.	Mkandawire E, Bisai C, Dyke E, Dressel A, Kantayeni H, Molosoni B, et al. A qualitative
536 assessment of gender roles in child nutrition in Central Malawi. BMC Public Health. 2022;22(1):1392.
537 45.	Grundy E, Kravdal Ø. Do short birth intervals have long-term implications for parental health?
538 Results from analyses of complete cohort Norwegian register data. Journal of Epidemiology and 539	Community Health. 2014;68(10):958.
540 46.	Hawkes C, Fanzo J. Nourishing the SDGs: Global nutrition report 2017. 2017.
541 47.	Nations FaAOotU. Integrating Agriculture and Nutrition Education for Improved Young Child
542 Nutrition. Rome: Food and Agriculture Organisation; 2016.
543 48.	Malawi Go. Nutrition Handbook for Farmer Field Schools. Lilongwe: Ministry of Agriculture,
544 Irrigation and Water Development; 2015.
545 49.	Canavan CR, Graybill L, Fawzi W, Kinabo J. The SDGs Will Require Integrated Agriculture,
546 Nutrition, and Health at the Community Level. Food Nutr Bull. 2016;37(1):112-5.
547 50.	Mkandawire E, Hendriks SL. A qualitative analysis of men’s involvement in maternal and child
548 health as a policy intervention in rural Central Malawi. BMC Pregnancy and Childbirth. 2018;18(1):37. 549

550

551

552

Figure 2	[Click here to access/download;Figure;Fig 2 Gender Norms Contributing to Stunting.docx](https://www.editorialmanager.com/pone/download.aspx?id=33649265&guid=410d45ca-d6f9-43d5-9a85-e68f3f07d87f&scheme=1)

|  |  | **Domains of RinGS Framework for Gender Analysis** | **Corresponding Theme** | **Categories** |
| --- | --- | --- | --- | --- |
|  |  |  | Gender unequal decision making on the consumption, sale and distribution of food | Distinction of roles in IYCF |
|  |  | *Division of labour, roles and everyday practices* |  | Competing spending needs |
|  |  |  |  | Nutrition not a priority in household spending |
|  |  | *Rules and decision making* |  | Men control decisions on use of farm produce |
| **How gender**  **norms affect stunting in Ntchisi district, Malawi** |  |  |  |  |
|  |  |  |  |  |
|  |  |  |  | Mothers at health centres, fathers at home |
|  |  | *Social norms and values* | Enshrined community norms influence feeding practices underpinned by gender based violence | Household conflict and gender based violence |
|  |  |  |  | Women accept GBV: fear and avoid shame |
|  |  |  |  | Prevalence of GBV after harvesting time |
|  |  |  |  | Men’s status in the community an enduring norm |
|  |  | *Access to resources* | Policy disconnections and gaps that reinforce unequal gender norms at the structural level | Agriculture, nutrition interventions disconnected |
|  |  |  |  | Poor male involvement in nutrition interventions |
|  |  |  |  | Teen motherhood and large family sizes |
|  |  |  |  | Low education and literacy levels |

Figure 3	[Click here to access/download;Figure;Fig 3 Factors Contributing to Stunting UNICEF.docx](https://www.editorialmanager.com/pone/download.aspx?id=33649282&guid=4e5cb904-fcbd-4282-bdb2-8e8e24868d10&scheme=1)

Figure 1	[Click here to access/download;Figure;Fig 1 UNICEF Framework of Malnutrition.docx](https://www.editorialmanager.com/pone/download.aspx?id=33649382&guid=c755e1e7-a40c-449c-a8d8-5b79994cc376&scheme=1)

**Source**: Reinhardt K, Fanzo J. Addressing Chronic Malnutrition through Multi-Sectoral, Sustainable Approaches: A Review of the Causes and Consequences. Front Nutr. 2014;1:13.
