# Supplementary material for: How do gender norms contribute to stunting in Ntchisi District, Malawi? a qualitative study
Source: PLoS One. 2024 Oct 22;19(10):e0290199. doi: 10.1371/journal.pone.0290199 (PMC11495627; doi:10.1371/journal.pone.0290199)
Supplement: Attachment — Submitted filename: R1_PLOSONE Response to Reviewers (1).docx [file pone.0290199.s003.docx]

| **REVIEWERS COMMENTS** | **REQUIRED ACTION** | **AUTHOR’S RESPONSE** |
| --- | --- | --- |
| **R1** |  |  |
| Congratulations to the authors. The paper is an interesting read and is well written in a scientific manner. The paper addresses an important subject matter of malnutrition and gender roles are indeed a mater issues that needs to be addressed |  | Thank you for your time and consideration in reviewing this piece. We appreciate your feedback and believe that the manuscript is greatly improved as a result of the reviewer comments. Thank you again for your time in reviewing this. |
| **R2** |  |  |
| In the abstract, the study appears not to have target population. |  | Thank you for this feedback. At community level, the target population was fathers and mothers at household level. At district/implementation level, the target population were the coordinating committees for nutrition and agriculture. At health management level, the target population was the NRU for health management. These have now been summarized and reported in the abstract under research methods. |
| Lines 48& 49 Defined by the World Health Organization (WHO) as “deficiencies, excesses or imbalances in a person’s intake of energy and/or nutrients | ”- What was defined by WHO? The statement needs to be reconstructed. | Thank you for this observation, we have amended this. |
| Line 112 While reduced from 47% in 2010,(29) the prevalence of stunting in children under-five years in……………….. | This phrase might not be appropriate to start a paragraph, please kindly re-phrase | Thank you for your observation and feedback. This statement has been rephrased. It is now on line 116. |
| Line 113. Among one of the most poorly………………… | You might need to delete among | Thank you for this observation, we have amended this. This phrase is now on line 119. |
| Lines 181-183 Ethics approval was granted by the Trinity Health Policy and Management, Centre for Global Health (HP&M/CGH) Research Ethics Committee (REC) and by the Ntchisi District Council Secretariat, including the District Health Office (DHO) and the District Agriculture Office (DAO) | Insert the study’s ethical approval number | Reference Numbers have been added for both RECs. |
| Informed verbal…………… |  | Thank you for this observation, we have amended this. |
| Line 199 a mean age of 26 while fathers had a mean age of 38. Participants reported………… | Mean must be reported with its SD value | This information was gleaned from the focus group discussions rather than calculated based on the use of a demographic questionnaire. We have amended the language to reflect this in the manuscript. |
| Lines 200 & 201 Most mothers and fathers were educated to primary level only, with some to second level. | What’s the percentage figure?  Percentage should also be reported to quantify the difference | Thank you. This information was also gleaned from the brief introductions by the participants at the outset of the focus group discussions. The manuscript has been amended to reflect this on line 202. |
| Line 332 Discussion and Conclusion | Conclusion must be separate as contained in the author’s guide for this journal | Thank you for noting this. This has been amended. |
| Kindly insert the DOIs for the cited articles, and effect all necessary corrections by providing the missing information in the references |  | Thank you for this feedback. This has been amended as reflected in the references section. The Vancouver style as stipulated by PLOS ONE does not include DOIs. We have adjusted to another numeric style, JAMA, which does include the DOIs. |
| The topic and the discuss appears not to tally, there are no information on feeding pattern of the children and their nutritional status. |  | Thank you for this feedback. The discussion focused on the three main themes in the findings; decision-making around production and consumption, norms around feeding practices and the policy gaps fostering gender norms as fully presented in the results section. The research did not focus discussion around feeding patterns as children were accessed through growth monitoring sessions. These sessions screen children for malnutrition; on which data was already presented demonstrating that the levels of malnutrition are generally high in this area. The absence of information on feeding patterns has been added as a limitation in this study and a recommendation for future studies that research is conducted at household level to capture feeding patterns. |
| Secondly, is the study a purposive sampling of parents with stunted children? |  | Thank you for this observation. This study purposively targeted mothers and fathers at growth monitoring sessions in an area where children were generally stunted, as well as coordinating/management level committees. The first author was a district nutritionist and was familiar with the general experience of under-nutrition as captured through the growth monitoring sessions. |
| Thirdly, there is no correlational study to show the impact of gender norm on children’s nutritional status, so the study appears to be inconclusive. |  | We appreciate this feedback and the time you took to review this document.  This study was a qualitative investigation of the way in which gender roles shape access to resources and impact child stunting. Rather than defining and measuring impact, qualitative studies seek to highlight the processes by which particular phenomena occur and the meaning ascribed to that phenomena by participants.  We have added the lack of correlation as a limitation and recommended that future studies should seek to examine this. |
