# Supplementary material for: How do gender norms contribute to stunting in Ntchisi District, Malawi? a qualitative study
Source: PLoS One. 2024 Oct 22;19(10):e0290199. doi: 10.1371/journal.pone.0290199 (PMC11495627; doi:10.1371/journal.pone.0290199)
Supplement: Attachment — Submitted filename: PONE-D-23-21165_R1_reviewer.docx [file pone.0290199.s004.docx]

PLOS ONE

**How do gender norms contribute to stunting in Ntchisi District, Malawi? A qualitative study**

--Manuscript Draft--

| **Manuscript Number:** | PONE-D-23-21165R1 |
| --- | --- |
| **Article Type:** | Research Article |
| **Full Title:** | How do gender norms contribute to stunting in Ntchisi District, Malawi? A qualitative study |
| **Short Title:** | Gender norms and stunting in Ntchisi, Malawi. |
| **Corresponding Author:** | Frédérique Vallières, Ph.D.  Trinity College Dublin: The University of Dublin Trinity College Dublin, IRELAND |
| **Keywords:** | stunting; gender norms; Malnutrition; Malawi; Children; UNICEF Conceptual Framework for Malnutrition |
| **Abstract:** | Background and Aim  Despite adequate food production and nutrition intervention coverage, stunting remains an enduring problem in Ntchisi, Malawi. Globally, gender and social norms are known to influence nutritional outcomes in children. This study explores how gender norms contribute to child stunting, in Ntchisi district, Central Malawi.  Research Methods  Informed by the UNICEF Framework for Malnutrition, nine focus group discussions were conducted with mothers (n=24), fathers (n=23), and members of policy and health treatment committees (n=21), spanning three different areas of Ntchisi district. Data were analysed through inductive thematic analysis, guided by the framework for Research in Gender and Ethics (RinGs).  Results  Three primary themes were identified: 1) gender unequal decision making on the consumption, sale and distribution of food; 2) enshrined community norms influence feeding practices underpinned by gender-based violence; and 3) policy disconnections and gaps that reinforce gender norms regarding nutrition. Themes encompassed practices across household, health treatment, and policy level.  Conclusion  Gender norms that underpin inequalities in decision making for production and consumption of food undermine children’s nourishment and contributes towards sustained child malnutrition in Ntchisi. Existing policy documents should revise their guidelines to incorporate gender norms as key determinants of malnutrition. |
| **Order of Authors:** | Whitney Mphangwe, MSc |
|  | Ann Nolan, PhD |
|  | Frédérique Vallières, PhD |
|  | Mairéad Finn, PhD |
| **Response to Reviewers:** | REVIEWERS COMMENTS  Reviewer 1: Congratulations to the authors. The paper is an interesting read and is well written in a scientific manner. The paper addresses an important subject matter of malnutrition and gender roles are indeed a mater issues that needs to be addressed  Respnse: Thank you for your time and consideration in reviewing this piece. We appreciate your feedback and believe that the manuscript is greatly improved as a |

|  | result of the reviewer comments. Thank you again for your time in reviewing this.  Reviewer 2  Reviewer 2; In the abstract, the study appears not to have target population. Response: Thank you for this feedback. At community level, the target population was fathers and mothers at household level. At district/implementation level, the target population were the coordinating committees for nutrition and agriculture. At health management level, the target population was the NRU for health management. These have now been summarized and reported in the abstract under research methods.  Reviewer 2: Lines 48& 49 Defined by the World Health Organization (WHO) as “deficiencies, excesses or imbalances in a person’s intake of energy and/or nutrients”- What was defined by WHO? The statement needs to be reconstructed.  Response: Thank you for this observation, we have amended this.  Reviewer 2: Line 112 While reduced from 47% in 2010,(29) the prevalence of stunting in children under-five years in… This phrase might not be appropriate to  start a paragraph, please kindly re-phrase  Response: Thank you for your observation and feedback. This statement has been rephrased. It is now on line 116.  Reviewer 2: Line 113. Among one of the most poorly… You might need to  delete among  Response: Thank you for this observation, we have amended this. This phrase is now on line 119.  Reviewer 2: Lines 181-183 Ethics approval was granted by the Trinity Health Policy and Management, Centre for Global Health (HP&M/CGH) Research Ethics Committee (REC) and by the Ntchisi District Council Secretariat, including the District Health Office (DHO) and the District Agriculture Office (DAO)Insert the study’s ethical approval number  Response: Reference Numbers have been added for both RECs.  Reviewer 2: Informed verbal… Thank you for this observation, we have  amended this.  Reviewer 2: Line 199 a mean age of 26 while fathers had a mean age of 38. Participants reported… Mean must be reported with its SD value  Response: This information was gleaned from the focus group discussions rather than calculated based on the use of a demographic questionnaire. We have amended the language to reflect this in the manuscript.  Reviewer 2: Lines 200 & 201 Most mothers and fathers were educated to primary level only, with some to second level.What’s the percentage figure?  Percentage should also be reported to quantify the difference  Response: Thank you. This information was also gleaned from the brief introductions by the participants at the outset of the focus group discussions. The manuscript has been amended to reflect this on line 202.  Reviewer 2: Line 332 Discussion and ConclusionConclusion must be separate as contained in the author’s guide for this journal  Response: Thank you for noting this. This has been amended.  Reviewer 2: Kindly insert the DOIs for the cited articles, and effect all necessary corrections by providing the missing information in the references  Response: Thank you for this feedback. This has been amended as reflected in the references section. The Vancouver style as stipulated by PLOS ONE does not include DOIs. We have adjusted to another numeric style, JAMA, which does include the DOIs.  Reviewer 2: The topic and the discuss appears not to tally, there are no information on feeding pattern of the children and their nutritional status. |
| --- | --- |

|  | Response: Thank you for this feedback. The discussion focused on the three main themes in the findings; decision-making around production and consumption, norms around feeding practices and the policy gaps fostering gender norms as fully presented in the results section. The research did not focus discussion around feeding patterns as children were accessed through growth monitoring sessions. These sessions screen children for malnutrition; on which data was already presented demonstrating that the levels of malnutrition are generally high in this area. The absence of information on feeding patterns has been added as a limitation in this study and a recommendation for future studies that research is conducted at household level to capture feeding patterns.  Reviewer 2: Secondly, is the study a purposive sampling of parents with stunted children?  Response: Thank you for this observation. This study purposively targeted mothers and fathers at growth monitoring sessions in an area where children were generally stunted, as well as coordinating/management level committees. The first author was a district nutritionist and was familiar with the general experience of under-nutrition as captured through the growth monitoring sessions.  Reviewer 2: Thirdly, there is no correlational study to show the impact of gender norm on children’s nutritional status, so the study appears to be inconclusive.  Response: We appreciate this feedback and the time you took to review this document. This study was a qualitative investigation of the way in which gender roles shape access to resources and impact child stunting. Rather than defining and measuring impact, qualitative studies seek to highlight the processes by which particular phenomena occur and the meaning ascribed to that phenomena by participants. We have added the lack of correlation as a limitation and recommended that future studies should seek to examine this. |
| --- | --- |
| **Additional Information:** |  |
| **Question** | **Response** |
| **Financial Disclosure**  Enter a financial disclosure statement that describes the sources of funding for the work included in this submission. Review the [submission guidelines](http://journals.plos.org/plosone/s/submission-guidelines#loc-financial-disclosure-statement) for detailed requirements. View published research articles from [*PLOS ONE*](http://journals.plos.org/plosone/) for specific examples.  This statement is required for submission and **will appear in the published article** if the submission is accepted. Please make sure it is accurate. | The author(s) received no specific funding for this work. |

| **Unfunded studies**  Enter: *The author(s) received no specific funding for this work.*  **Funded studies**  Enter a statement with the following details:   - Initials of the authors who received each award - Grant numbers awarded to each author - The full name of each funder - URL of each funder website - Did the sponsors or funders play any role in the study design, data collection and analysis, decision to publish, or preparation of the manuscript? - **NO** - Include this sentence at the end of your statement: *The funders had no role in study design, data collection and analysis, decision to publish, or preparation of the manuscript.* - **YES** - Specify the role(s) played. |  |
| --- | --- |
| * typeset |  |
| **Competing Interests**  Use the instructions below to enter a competing interest statement for this submission. On behalf of all authors, disclose any [competing interests](http://journals.plos.org/plosone/s/competing-interests) that could be perceived to bias this work—acknowledging all financial support and any other relevant financial or non- financial competing interests.  This statement is required for submission and **will appear in the published article** if the submission is accepted. Please make sure it is accurate and that any funding sources listed in your Funding Information later in the submission form are also declared in your Financial Disclosure statement.  View published research articles from  [*PLOS ONE*](http://journals.plos.org/plosone/) for specific examples. | The authors have declared that no competing interests exist. |

| **NO authors have competing interests**  Enter: *The authors have declared that no competing interests exist.*  **Authors with competing interests**  Enter competing interest details beginning with this statement:  *I have read the journal's policy and the authors of this manuscript have the following competing interests: [insert competing interests here]* |  |
| --- | --- |
| * typeset |  |
| **Ethics Statement**  Enter an ethics statement for this submission. This statement is required if the study involved:   - Human participants - Human specimens or tissue - Vertebrate animals or cephalopods - Vertebrate embryos or tissues - Field research   Write "N/A" if the submission does not require an ethics statement.  General guidance is provided below. Consult the [submission guidelines](http://journals.plos.org/plosone/s/submission-guidelines#loc-guidelines-for-specific-study-types) for detailed instructions. **Make sure that all information entered here is included in the Methods section of the manuscript.** | Ethics approval was granted by the Trinity Health Policy and Management, Centre for Global Health (HP&M/CGH) Research Ethics Committee (REC) and by the Ntchisi District Council Secretariat, including the District Health Office (DHO) and the District Agriculture Office (DAO). Informed consent was sought from participants, who were fully briefed about the study aims and objectives, the voluntary nature of their participation, and that they could opt out at any time without penalty. |

| **Format for specific study types**  **Human Subject Research (involving human participants and/or tissue)**   - Give the name of the institutional review board or ethics committee that approved the study - Include the approval number and/or a statement indicating approval of this research - Indicate the form of consent obtained (written/oral) or the reason that consent was not obtained (e.g. the data were analyzed anonymously)   **Animal Research (involving vertebrate animals, embryos or tissues)**   - Provide the name of the Institutional Animal Care and Use Committee (IACUC) or other relevant ethics board that reviewed the study protocol, and indicate whether they approved this research or granted a formal waiver of ethical approval - Include an approval number if one was obtained - If the study involved *non-human primates*, add *additional details* about animal welfare and steps taken to ameliorate suffering - If anesthesia, euthanasia, or any kind of animal sacrifice is part of the study, include briefly which substances and/or methods were applied   **Field Research**  Include the following details if this study involves the collection of plant, animal, or other materials from a natural setting:   - Field permit number - Name of the institution or relevant body that granted permission |  |
| --- | --- |
|  |  |
| **Data Availability**  Authors are required to make all data underlying the findings described fully available, without restriction, and from the time of publication. PLOS allows rare exceptions to address legal and ethical concerns. See the [PLOS Data Policy](http://journals.plos.org/plosone/s/data-availability) and [FAQ](http://journals.plos.org/plosone/s/data-availability#loc-faqs-for-data-policy) for detailed information. | Yes - all data are fully available without restriction |

| A Data Availability Statement describing where the data can be found is required at submission. Your answers to this question constitute the Data Availability Statement and **will be published in the article**, if accepted. |  |
| --- | --- |
| **Important:** Stating ‘data available on request from the author’ is not sufficient. If your data are only available upon request, select ‘No’ for the first question and explain your exceptional situation in the text box. |  |
| Do the authors confirm that all data underlying the findings described in their manuscript are fully available without restriction? |  |
| **Describe where the data may be found in full sentences. If you are copying our sample text, replace any instances of XXX with the appropriate details.**   - If the data are **held or will be held in a public repository**, include URLs, accession numbers or DOIs. If this information will only be available after acceptance, indicate this by ticking the box below. For example: *All XXX files are available from the XXX database (accession number(s) XXX, XXX.*). - If the data are all contained **within the manuscript and/or Supporting Information files**, enter the following: *All relevant data are within the manuscript and its Supporting Information files.* - If neither of these applies but you are able to provide **details of access elsewhere**, with or without limitations, please do so. For example:   *Data cannot be shared publicly because of [XXX]. Data are available from the XXX Institutional Data Access / Ethics Committee (contact via XXX) for researchers who meet the criteria for access to confidential data.*  *The data underlying the results presented in the study are available from (include the name of the third party* | Pseudonymised transcripts will be made available in Zenodo effective from date of publication: 10.5281/zenodo.11478993 |

| *and contact information or URL).*   - This text is appropriate if the data are owned by a third party and authors do not have permission to share the data.   * typeset |  |
| --- | --- |
| Additional data availability information: | Tick here if the URLs/accession numbers/DOIs will be available only after acceptance of the manuscript for publication so that we can ensure their inclusion before publication. |

Manuscript	[Click here to access/download;Manuscript;R1 Unmarked Manuscript_Stunting_UNICEF_Gender_Malawi - Copy.docx](https://www2.cloud.editorialmanager.com/pone/download.aspx?id=36521522&guid=3c464831-f5b3-44e4-9198-31f5fcc36750&scheme=1)

# 1 How do gender norms contribute to stunting in Ntchisi District, Malawi? A qualitative study

2 Whitney Mphangwe^1*,2^, Ann Nolan^1^, Frédérique Vallieres^2^, Mairéad Finn^3,4^
3 [mphangww@tcd.ie](mailto:mphangww@tcd.ie); [fvallier@tcd.ie](mailto:fvallier@tcd.ie); [nolana13@tcd.ie](mailto:nolana13@tcd.ie); [finnm2@tcd.ie](mailto:finnm2@tcd.ie)
4 ^1^ Trinity Centre for Global Health, School of Medicine, Trinity College Dublin
5 ^2^ Food and Nutrition Officer, Ministry of Agriculture, Irrigation and Water Development
6 ^3^ Trinity Centre for Global Health, School of Psychology, Trinity College Dublin.
7 ^4^ Department of Public Health and Epidemiology, Royal College of Surgeons in Ireland
8 *Corresponding Author 9

10

11 Whitney Mphangwe
12 Ministry of Agriculture, Irrigation and Water Development
13 Ntchisi District Agriculture Office
14 P.O. Box 37
15 Ntchisi
16 Malawi 17
18 Mairéad Finn
19 Centre for Global Health, University of Dublin, Trinity College
20 7 – 9 Leinster Street South
21 Dublin 2
22 Ireland 23

# 24 How do gender norms contribute to stunting in Ntchisi District, Malawi? A qualitative study

25 **Abstract**
26 **Background and Aim:** Despite adequate food production and nutrition intervention coverage,
27 stunting remains an enduring problem in Ntchisi, Malawi. Globally, gender and social norms are known
28 to influence nutritional outcomes in children. This study explores how gender norms contribute to
29 child stunting, in Ntchisi district, Central Malawi.
30 **Research Methods:** Informed by the UNICEF Framework for Malnutrition, nine focus group
31 discussions were conducted with a target population of mothers (n=24), fathers (n=23) purposively
32 targeted through growth monitoring sessions, and members of policy and health treatment
33 committees (n=21), spanning three different areas of Ntchisi district. Data were analysed through
34 inductive thematic analysis, guided by the framework for Research in Gender and Ethics (RinGs).
35 **Results:** Three primary themes were identified: 1) gender unequal decision making on the
36 consumption, sale and distribution of food; 2) enshrined community norms influence feeding practices
37 underpinned by gender-based violence; and 3) policy disconnections and gaps that reinforce gender
38 norms regarding nutrition. Themes encompassed practices across household, health treatment, and
39 policy level.
40 **Conclusion:** Gender norms that underpin inequalities in decision making for production and
41 consumption of food undermine children’s nourishment and contributes towards sustained child
42 malnutrition in Ntchisi. Existing policy documents should revise their guidelines to incorporate gender
43 norms as key determinants of malnutrition.
44 **Keywords:**	Stunting, Gender Norms, Malnutrition, Malawi, Children, UNICEF Conceptual
45 Framework for Malnutrition

# 46 How do gender norms contribute to stunting in Ntchisi District, Malawi? A qualitative study

47 **Introduction**
48 The World Health Organization (WHO) defines malnutrition as “deficiencies, excesses or imbalances
49 in a person’s intake of energy and/or nutrients”^1^ broadly incorporating under-nutrition, micro-
50 nutrient related malnutrition, and over-nutrition. Malnutrition remains a topic of global concern, with
51 20% of the global under-five population (149.2 million) currently ‘stunted,’ that is, a low height-for-
52 age index,^2^ and where malnutrition accounts for 53% of under-5 mortality.^3^ Stunting causes long-term
53 impairment including poor school performance in childhood, reduced productivity later in life, and an
54 increased risk of chronic non-communicable illnesses; and, in the worst cases, death.^4^
55 Numerous international policy commitments seek to address malnutrition. The United Nations
56 (UN) Sustainable Development Goals (SDGs), particularly SDG2, aspire to improve agriculture and food
57 systems to feed and nourish malnourished populations.^5^ Likewise, the WHO’s 2018 Global Nutrition
58 Target (GNT) sought to reduce the number of stunted children by 40%^6^ and the UN’s 2015 General
59 Assembly declared 2015-2025 to be the decade of nutrition, a commitment by member states to
60 implement policies, programs, and investments to eliminate malnutrition in all its forms. Scaling Up
61 Nutrition (SUN) – a global movement working with the UN, civil society, business and donor networks
62 to tackle malnutrition - promotes exclusive care and investment in child care globally, particularly in
63 the first 1000 days of life.^7^
64 Established determinants of malnutrition include poor maternal nutrition and young child
65 feeding practices;^8,9^ young mothers’ age at first birth, which is linked to higher parity and poorer birth
66 spacing practices;^10^ depleted nutrition for mothers experiencing successive pregnancies and
67 lactation,^8^ associated with poorer feeding practices;^11^ subclinical infections from exposures to
68 contaminated environments;^12,13^ and infectious diseases such as malaria, which can compound short
69 term loss of weight and contribute to long term stunting.^14^ Malnutrition is thus determined by the
70 complex interaction of multiple social, economic, and cultural factors. These factors are reflected in
71 UNICEF’s Conceptual Framework of Maternal and Child Nutrition (Figure 1),^15^ often considered the
72 primary global policy instrument to address the causes and consequences of malnutrition and reduce
73 its prevalence.^16^ According to this framework, *basic-level* causes operate in the broader context and
74 include socio-economic and cultural context, human capital, and access to potential resources;
75 *underlying* causes operate at household and community level, including care and feeding practices;
76 and *immediate* causes relate to direct diet intake and the presence of disease.^16^ Recently updated in
77 2020, the framework now also recognizes contributors to good nutrition as being either immediate,
78 underlying, or enabling determinants.^17^

# 79 Figure 1: UNICEF Framework for Malnutrition

80 [INSERT FIGURE ONE HERE]

81

82 Gender refers to the socially constructed roles, behaviors, activities, and attributes that a given
83 society considers appropriate for men and women.^18^ More recently, growing attention has been
84 brought to the impact of gender norms on child malnutrition and stunting.^19^ In many settings, women
85 still cannot exercise freedom and autonomy of choice, including within their own households,^14^ with
86 the extent to which a mother can make independent decisions negatively associated with the
87 likelihood of childhood stunting.^19^ Similarly, granting women greater decision-making power is
88 associated with notable improvements in their children’s height-for-age index.^20^ Likewise, analysis of
89 gender relations in norms and decision-making has recently come to the fore in explaining the
90 endurance of problematic health outcomes.^21^ Indeed, positive social and cultural norms (which
91 include gender) are recognized as one of the most enabling determinants of improved nutrition for
92 women and children.^17^
93 In recognition of the role of gender and social norms in determining nutritional outcomes in
94 children, several frameworks have been created to guide gender analyses within health and nutritional
95 programming,^22-24^ which differ in their focus on types of power or area of the health system.
96 Specifically, the Research in Gender and Ethics (RinGS) Framework articulates gender differences as
97 unfolding across four domains: access to resources; division of labour, roles, and everyday practices;
98 social norms and values; and rules and decision making.^25,26^ It is considered comprehensive for
99 examining social and structural elements of gender interacting with health.^21^

# 100 Malnutrition in Malawi

101 Categorized as one of the poorest countries in the world by the World Bank,^27^ Malawi is a Southern
102 African country home to over 18 million inhabitants. A signatory to the SUN movement since 2011,
103 the Malawian Government applies UNICEF’s Conceptual Framework for Malnutrition (1990) to
104 address malnutrition.^16^ Moreover, the Malawi Multi-Sectoral National Nutrition Policy (MSNNP) sets
105 out the country’s priority areas for implementing nutrition interventions across the country.^28^
106 Specifically, these priority areas include (i) the prevention of under-nutrition, (ii) achieving gender
107 equality, equity protection, participation and empowerment, and (iii) the treatment and control of
108 malnutrition.^28^ The MSNNP outlines best practices to end malnutrition, detailing policy and
109 implementation plans to address causes of malnutrition at household, health treatment, and policy
110 levels.^28^
111 Malawi has registered some reduction in the prevalence of stunting among children under five
112 from 47% in 2010,^29^ to 37% in 2015 (from the most recent available data), which is still very high. One
113 of the most poorly affected districts in the country is Ntchisi, where 40% of the population are
114 malnourished.^29^ Paradoxically, Ntchisi is also considered one of the most household food secure
115 districts in the country.^30,31^ Being food secure defined as people having at all times physical, social and
116 economic access to food, safely consumed in sufficient quantity and quality for dietary needs and food
117 preferences, supported by an environment of adequate water and sanitation, health services and
118 care.^32,33^ Furthermore, exclusive breastfeeding rates in Ntchisi district for infants under six months
119 (61%) exceed the global average (40%).^29,2^
120 Evidence suggests that gender norms may play a role in Ntchisi’s observed malnutrition rates.
121 For example, in 2016, 47% of women were married before 18 years and 29% of adolescent girls were
122 mothers.^29,34^ Women were also found to be five times less likely to make independent decisions when
123 compared with men, a difference linked to variation in earning power.^35^ There is evidence that
124 differences in gender norms have a detrimental impact on women’s health.^36^ *How* differences in
125 gender norms may contribute to malnourishment and stunting in children, in Ntchisi, Malawi,
126 however, remain unexplored. This study therefore aimed to explore how gender norms play a role in
127 continued stunting in Ntchisi District, Malawi, and to contribute to a revised version of the UNICEF
128 Framework for Malnutrition to better reflect gender norms.
129 129

# 130 Materials and Methods

131 *Design and Sampling*
132 We employed a qualitative design, as part of a wider examination of the causes of observed stunting
133 in Ntchisi district. We conducted nine focus group discussions (FGDs) to explore the role of gender
134 norms in persistent child stunting across three different communities. FGDs were selected as they are
135 considered valuable for generating data on group social norms, while also prioritising participants’
136 perspectives.^37^ Each community was purposely selected to achieve geographical spread. Six FGDs -
137 two in each community - were conducted with mothers and fathers as household members, one FGD
138 was conducted with participants delivering nutrition health treatment and two were conducted with
139 participants working on nutrition policy. Therefore, nine focus groups explored how gender norms
140 might impact (mal)nutrition, reflecting household, health treatment and nutrition policy spheres as
141 articulated in the Malawi MSNNP.^28^
142 In total, 68 participants were recruited, with 6-9 people represented within each FGD. The six
143 FGDs with household members comprised three with males (n=24) and three with females (n=23).
144 Females were within the reproductive age group, had at least one child under the age of five in the
145 household, and were recruited through Growth Monitoring Sessions. There was no age limitation for
146 males, who were also required to have at least one child under the age of five in the household. Men
147 were recruited from nearby social gathering venues, mostly at church choir gatherings and market
148 points via word-of-mouth by the government extension workers.
149 At health treatment level, participants (n=10) included five females and five males of the
150 Nutrition Rehabilitation Unit (NRU) Management Committee. This committee comprises nutritionist,
151 nurse, and home-help workers providing treatment to severe cases of malnutrition admitted to the
152 District Health Office. Policy level participants (n=10) also comprised five females and five males of
153 the District Agriculture Coordinating Committee (DAECC), a committee that oversees and coordinates
154 all agricultural interventions at the district level, and the District Nutrition Coordinating Committee
155 (DNCC), a committee responsible for coordinating all nutrition interventions at the district.
156 156
157 Data Collection Instruments
158 Semi-structured focus group discussion guides were developed with reference to the domains of the
159 UNICEF Framework of Malnutrition^16,17^, incorporating probes to ensure maximum investigation of
160 persistent stunting. The guides included general questions about daily activities, food and income;
161 resources and decision-making; and understanding of and perspectives on stunting. Discussions were
162 conducted by the first author in Chichewa, the local language. Two field assistants, one from the health
163 sector and the other from the agriculture sector, took notes of the participants’ answers throughout
164 the discussions as a backup for the audio recordings.
165 165
166 Data Management and Analysis
167 FGDs were recorded, transcribed verbatim,^38^ and translated from Chichewa to English with close
168 reference to audio files and notes. All personal identifiers were removed. Following a thorough review
169 of the transcripts for familiarity, analysis comprised three phases of open (i.e. inductive) coding,
170 categorization, and abstraction. Transcripts were coded according to the steps of Braun and Clarke^39^,
171 with common phrases assigned codes, then inductively organized into categories and themes. A
172 framework for gender analysis,^25,26^ informed by Research in Gender and Ethics (RinGs)^22^ guided
173 analytic insights. Interpretation of the data was enhanced by discussions between the first author,
174 the field workers, and the second author to refine analytical development and verify the conceptual
175 coherence of the codes.
176 176
177 Ethics, Validity and Reliability
178 Ethics approval was granted by the Trinity Health Policy and Management, Centre for Global Health
179 (HP&M/CGH) Research Ethics Committee (REC) (Ref. 20/2029/01) and by the Ntchisi District Council
180 Secretariat (Ref 2019/01/01). Informed verbal consent was sought from participants, who were fully
181 briefed about the study aims and objectives, the voluntary nature of their participation, and that they
182 could opt out at any time without penalty.
183 The validity and reliability of the data was ensured through transcription, clear and transparent
184 coding, reflexivity, and cross-checking of themes between the lead author, the second author, and
185 two field assistants.^40^ The first author, who carried out the data collection, is a nutritionist in Ntchisi
186 District, and reflexive attention was paid to the influence of her positionality throughout the data
187 collection and analysis phases.
188 188
189 189

# 190 Results:

191 **Participant Characteristics**
192 Discussions commenced with a brief introduction from all participants. From this, it was gleaned that
193 female participants ranged between 18 to 38 years of age and males ranged from 25 to 56 years of
194 age. Participants reported having five children, on average. From the introductions, it was also gleaned
195 that most mothers and fathers were educated to primary level only, with some to second level.
196 Agriculture was their main source of food and income. Among committee members, half were
197 employed by government while the other half were employed by non-government organizations.

# 198 Exploring the Role of Gender Norms in Contributing to Malnutrition

199 Three core themes were identified in the data to illuminate the role of gender norms in contributing
200 to malnutrition and stunting in Ntchisi. These were: (i) gender unequal decision making on the
201 consumption, sale and distribution of food; (ii) enshrined community norms influence feeding
202 practices, underpinned by gender based violence; (iii) policy disconnections and gaps that reinforce
203 unequal gender norms regarding nutrition. Themes are presented in Figure 2, aligned to the RinGS
204 Framework for Gender Analysis.^21,22^

| 205 |  |
| --- | --- |
| 206 | **Figure 2: Gender Norms Contributing to Stunting in Ntchisi District, Malawi** |
| 207 | [INSERT FIGURE TWO HERE] |
| 208 | **Gender Unequal Decision Making Shapes the Household Production and Consumption of Food** |
| 209 | Power differences between fathers and mothers affected decisions about how farm produce is |
| 210 | utilized. The policy level focus groups explained how practices in subsistence agriculture affect what |
| 211 | to produce, the quantity to produce, and where to sell produce, all of which affect how much remains |

212 for families to eat. Women in the district were reported to have lower decision-making power about
213 food production by comparison to men:
214 “Men dominate…they control what is cultivated and what to do with the produce. You find that
215 a woman might know that legumes are nutritious and essential for child growth… [but] when
216 the husband wants to sell, the [wife] will not resist.” DAECC FGD.
217 Describing their social context, household participants told of the different roles that mothers and
218 fathers take and how this impacts children’s nutrition. The participants recounted that within the
219 household, fathers are considered providers while mothers are nurturers. When describing their daily
220 routine, mothers mentioned household chores like cooking, feeding, laundry, tidying the house, and
221 collecting firewood and water, the latter usually from a great distance. On the other hand, fathers
222 reported daily work of gardening, casual tasks, socializing, and sourcing income for the household.
223 Fathers and mothers emphasised a clear distinction of roles in feeding children:
224 “Men search for food…we stay at home taking care of the children… cooking for and feeding
225 them. When men bring food, we cook.” Mothers FGD
226 “We take care of children in the sense that we search for food so that woman can cook it for
227 the children.” Fathers FGD.
228 However, fathers were characterized by participants as not health or nutrition minded in relation to
229 expenditure. Almost all mothers’ groups described that men in the community use their income on
230 pleasures rather than on actual needs.
231 “Sometimes, you can work hard to produce more. However, when you harvest and sell, the man
232 will spend it on drinking and other women. You cannot question how they spend it. They forget
233 about their own children. You face it all because the children usually stay at home with a
234 mother”. Mothers FGD.
235 Similarly, fathers indicated that when they harvest, they have several responsibilities to address.
236 Nutrition might not usually be the priority.
237 “A poor person always has a handful of things to take care of. When you get money…you don’t
238 remember what nutrition or nutritious food is about” Fathers FGD.
239 Differences between mothers and fathers in decision making about utilization of farm produce also
240 manifested at the health center. The health committee FGD recounted how in-patient cases of
241 malnutrition at the health centre are presented mostly by mothers, who attend with their children
242 while fathers stay at home. Extended stays can cause worry to mothers about further disruption to
243 home life and the oversale of food:
244 “The mothers wait for children here…we give them health talks every morning. Fathers usually
245 stay at home…[but]…when the mothers stay for some days, they start complaining thinking that
246 their husbands will marry another woman or get a chance to sell out farm produce and other
247 household assets. They just become so eager to go, and it is evident in how they act.” NRU FGD.
248 Although mothers in Ntchisi were primarily responsible for feeding children, they lacked decision-
249 making power over the production and consumption of food. Fathers, who held decision making
250 power, were not oriented towards nutritional needs for children. Furthermore, inequality in decision
251 making about food for children intensifies when women are not present.
252 252

# 253 Enshrined Community Norms Influence Feeding Practices, Underpinned by Gender Based Violence

254 Household focus groups reported that inequity in household decision making around food was
255 preserved by traditional norms of gender-based violence (GBV) in Ntchisi district. A clear and strong
256 description by participants was that women cooperate with men’s decisions for fear of GBV, and do
257 not speak out about GBV in fear of shaming their husbands and losing respect.
258 “In this area, when a woman has a brilliant idea, men do not want to appear cheap, so they do
259 not agree, whatever it is about…as a woman you just coil up and keep quiet otherwise you will
260 be beaten” Mothers FGDs
261 The supremacy of this social norm was bolstered by the status it confers on men in the community.
262 “Men here say things, and that is final…maybe it could be one out of ten men that would be
263 under petticoat government. If you allow your wife to be key in making decisions, your friends
264 will despise you…even young children would look down on you.” Fathers FGD.
265 District committee members recounted that cases of GBV are more prevalent at times of the year
266 when produce is sold. Gaps in power between mothers and fathers extended to a firm inequality in
267 autonomy, which impacted on children’s nutrition.
268 “Men are just bad. During the cultivating period, they work well with their wives, but when it is
269 selling time, they prefer to work alone and spend the money on drinking or other women…this
270 initiates conflicts that usually ends in abuse. Children are usually left with mothers that do not
271 have food supplies or money.” DNCC FGD.
272 Mothers and fathers highlighted that the clusters of friends and neighbours they lived among were
273 their primary sources of knowledge, indicating the strong traditional norms shaped how children were
274 taken care of. Gender based violent practices were encased in traditional community norms.
275 “Everybody does that…that’s how we do things here. Our parents teach us what they did so that
276 we grew up. When your child is born, they take you aside and teach you many things	we
277 apply that so that our children grow up too” Fathers FGD.
278 Thus, community adherence to traditional practice creates a challenge to do things differently
279 when feeding children. Rigid social norms of women required to be deferential to men were held in
280 place by traditional expectations and the threat of gender-based violence. These norms retained an
281 enduring social power in the community and impacted practices and decision making around food for
282 children.
283 283

# 284 Policy Disconnections and Gaps that Structurally Reinforce Gender Norms Regarding Nutrition

285 A disconnection between production and nutrition interventions was articulated by policy level
286 participants, with agricultural produce sold by subsistence farmers rather than retained for their
287 families. Participants explained that while the agriculture committee incorporates nutrition
288 interventions to its activities in theory; no nutrition achievements could be attributed to this work.
289 “We try to incorporate nutrition in our planning, but all detailed plans and achievements are
290 with the DNCC because we mainly focus on agriculture … however, nutrition is for DNCC, so
291 consult them for specific achievements.” DAECC FGD.
292 The disconnection indicates a wider aspect of the gender gap because agriculture interventions -
293 usually organized by men in the district - are implemented separately from general nutrition
294 interventions, targeting women as primary caregivers for children.
295 Men’s participation in the implementation of nutrition interventions was noted as a gap by
296 household level participants. Mothers encouraged targeting fathers for inclusion, highlighting that if
297 men were aware of nutrition issues, they would be more supportive at home and more informed
298 about nutrition-related decisions.
299 “Men should be taught. They are stubborn and irresponsible because they do not know and
300 understand. They should hear for themselves. They should be included in care groups. Now care
301 groups are only full of women” Mothers FGD
302 Despite father’s lack of involvement in nutrition reflecting the socio-cultural norms in Ntchisi,
303 men in the fathers’ focus groups indicated their willingness to participate in the sessions and learn
304 more about nutrition.
305 “The extension workers always teach women. The next training, please include us…we will come
306 and are happy to learn.” Fathers FGD
307 Impacts of trainings were also affected by education levels, specifically literacy, of the
308 population. Low levels of education were reported as a general feature of the population in Ntchisi,
309 affecting practices towards childcare. Health treatment level participants highlighted how literacy
310 levels affect the uptake of trainings.
311 “You can have a hospital, offer training in all these things…however, without school, it is all in
312 vain. You need some [education] to understand the importance of it all.” NRU FGD.
313 The Government’s role was further emphasized in reducing teen pregnancies and achievements
314 in nutrition education. Teen mothers face challenges in adequately nourishing themselves and their
315 children. Pressure is exacerbated when women have their first child at a young age, because it leads
316 to larger family size. Care demands of young children cannot be met if the mother has many children,
317 closely spaced together.
318 “I think the issue is that people have many children. You see some have eight, others nine. They
319 cannot afford to monitor if all ate or not. You would not know that one missed food because
320 they are too many..[…]… the Government should do more in reducing teen pregnancies…and
321 should invest in keeping girls in school.” NRU FGD.
322 Low levels of education undermine the household’s ability to meet the nutrition needs of children,
323 affecting both choices in health and well-being and the priority given to well-being. These trends are
324 also an indication of a broader structural context with unequal gender norms impacting women and
325 children.

# 326 Discussion

327 Despite a context of food security, interventions on nutritious feeding, and the development of
328 policies to target the eradication of undernutrition, children’s inadequate consumption of food in
329 Ntchisi is impacted by gendered practices and power dynamics. Specifically, differing roles between
330 men and women shape use of farm produce and feeding practices, enduring traditional social norms
331 preserved via the threat of gender-based violence and structurally bolstered through policy
332 disconnections, are all experienced as gender-related factors that impact on the nutrition of children
333 in Ntchisi.
334 Similar to Alemayehu et al.^14^, and Kamiya et al.^41^, women interviewed in Ntchisi district also had
335 limited power in making decisions about use of food within their households. Adhering to men’s
336 decisions regarding food production is a strong tradition that results in households being unable to
337 consume the right varieties and quantities. Although subsistence farming is generally characterized by
338 the consumption of produce,^42^ the narratives in this study highlighted how food produced for
339 subsistence is sold and distributed rather than consumed.	Subsistence farming also connotes
340 adherence to indigenous knowledge and traditions in production and consumption, pointing again to
341 the embedded nature of traditional practices.^43^
342 Power differences between women and men were also evident in that women were involved
343 in harvesting food, but excluded from decisions on the use of the harvest. Conflict related to decisions
344 on use of harvest produce reportedly resulted in increased gender-based violence or a rupture in the
345 marriage, as participants recounted risks of both increased violence and fathers sometimes
346 abandoning their families. This trend has been identified elsewhere, where women characterized
347 themselves as ‘harvest wives’ in cases where their husbands abandoned them when the harvest was
348 complete.^44^ A salient phenomenon that impacts the use of food is women’s lack of inclusion in
349 decisions on how harvested food will be used for the family, with a heightened risk of gender based
350 violence or relationship breakdown at this point.
351 While the government has an established role in imparting knowledge on food preparation and
352 feeding, enshrined gender norms undermine how women can practice their learning, as they are
353 pressured to adhere to men’s decisions. Women’s translation of their nutrition knowledge into
354 practice is also challenged by the high number of GBV cases reported in the district, reported as more
355 prevalent during harvest time, also contributing to the limited contribution of agricultural produce to
356 nourishment of children. Consistent with previous findings, children’s dietary intake in Ntchisi is also
357 reportedly further compromised by mothers’ young age and a large number of children, with
358 consequent reduced care practices.^45^ In the context where women do not make independent
359 decisions, it can be assessed that the lack of independence extends to decisions on family size and
360 birth control methods.
361 Father’s lack of involvement in their child’s nutrition within Ntchisi was also found to be a
362 manifestation of the social cultural norms in the district. Participants across the sample suggested
363 inclusion of men in community trainings on infant and young child feeding. In a social context where
364 fathers want to be respected for not being “under petticoat government”, the cultural change
365 required to incorporate men into nutrition interventions is evident. A more targeted approach to
366 addressing men’s roles in nutrition, that takes account of the bearing of gender norms, is required.
367 In support of the UNICEF Conceptual Framework of Malnutrition, the findings from this study
368 demonstrate that gender dynamics indeed shape malnutrition across all three causes of malnutrition
369 articulated in the framework - basic, underlying, and intermediate. Practices at the underlying levels
370 of household and community related to the production and consumption of food were highly unequal
371 for men and women. At the basic level of socio-economic and cultural context, capital, and access to
372 resources, powerful norms bolstering inequity between women and men were evident. Unequal
373 practices within these domains were highly gendered, intersecting to shape lack of access to and use
374 of resources for the nourishment of children. These findings and considerations are presented in
375 Figure Three as an expanded version of the UNICEF Conceptual Framework for Maternal and Child
376 Malnutrition.

# 377 Figure 3: Factors Contributing to Stunting adapted to the 1990 UNICEF Conceptual Framework of

378 **Malnutrition**
379 [INSERT FIGURE 3 HERE]
380 The current study adds to the existing literature by illustrating that in Ntchisi, gender norms
381 manifest across basic, underlying, and intermediate levels, and not just at the basic level as currently
382 articulated within the UNICEF conceptualization. Accordingly, gender should be articulated across the
383 entire UNICEF	framework, and	the	framework	might	benefit	from	applying	the	WHO’s
384 recommendations for incorporating intersectional gender analysis.^25,26^ In line with the domains within
385 RiNGs framework, domains of ‘access to resources’; ‘divisions of labour, roles and everyday practices’;
386 ‘social norms and values’; ‘rules and decision making’^26^ should also be incorporated into the UNICEF
387 framework to illuminate how unequal gender norms and roles unfold to explain continued problems
388 of nutrition for children.
389 The lack of policy coordination between agriculture and nutrition could contribute to
390 explanations of the disconnection between food production, food consumption, and nutrition status
391 in Ntchisi. Strengthening policy linkages between these domains would align with the 2017 Global
392 Nutrition Report^46^ statement that achieving good nutrition means practicing agriculture that feeds
393 and nourishes a nation state. The Food and Agriculture Organization emphasizes the importance of
394 integrating food production and nutrition interventions in parallel, and the findings presented here
395 provide insights for practical implementation.^47,48^ Integrating nutrition and agriculture is emphasized
396 by the theme of the 2016 World Food Day (WFD) commemorations; “our health is not only what we
397 eat, it is primarily what we grow”.^49^ Gender sensitivity must traverse these connections. Male
398 involvement in these connections is imperative, in recognition of the fact that they hold decision-
399 making power and asset ownership. Mkandawire & Hendricks (2015) note that while it is important
400 to target women and children in nutrition interventions, the exclusion of men ignores the fact that
401 women are limited in terms of decision-making powers and asset ownership.^50^
402 The Malawi MSNNP cites gender equality, equity protection, participation and empowerment^28^
403 as its second priority area. The findings can be translated into policy actions on under-nutrition and
404 stunting under the umbrella of the MSNNP. Specifically, government and development partners could
405 continue multi-sectoral implementation but place special focus on male involvement.
406 The Malawi MSNNP 2018-2022 emphasizes the need to involve all line ministries and
407 stakeholders in reducing child under-nutrition. In line with the policy objectives, this study
408 recommends that Government and other NGOs connect programmes across household, health
409 treatment and policy levels in Ntchisi; connect agriculture and nutrition polices; and incorporate a
410 gender lens. In addition, education must underpin all interventions, especially so for girls experiencing
411 teen pregnancy. The successful implementation of nutrition programmes relies on an educated and
412 literate population.
413 Conclusion
414 In conclusion, the findings of this study have demonstrated how (mal)nutrition is impacted by the
415 socially constructed roles, behaviours, activities and attributes that a given society considers
416 appropriate for men and women, which can manifest as unequal power relations shaping access to
417 and use of food.^26^ The unequal division of power and resources between men and women in Ntchisi
418 district in the central region of Malawi is a contributing factor to levels of malnutrition and stunting.
419 To address these influences on (mal)nutrition, global and national policy documents can integrate
420 gender sensitive approaches cutting across other policy areas, allowing gender to be fully integrated
421 in response. it appears appropriate to recommend that intersectional gender analysis that takes
422 account of gender inequality at household and community levels, should be mainstreamed into the
423 UNICEF framework as also recommended by the WHO. Efforts to improve child nutrition need to be
424 aligned with gender interventions to improve access, utilization and consumption of nutritious foods.
425 UNICEF’s conceptual	framework	of	malnutrition	should	highlight	gender	for	more	holistic
426 programming to reflect that improving the nutrition status of children in a highly productive
427 agricultural region is only possible if gender and nutrition interventions are indissolubly linked.

# 428 Limitations

429 The research had several limitations. Firstly, interviews were conducted in Chichewa, the vernacular
430 language in Ntchisi, and transcripts were translated to English by the first author who speaks Chichewa
431 and English fluently. This translation process, however, might have introduced some mis-explanations
432 of meanings of terms. Second, the first author is a nutrition officer who works in the area under study.
433 While every effort was made to account for the researcher role, it is challenging to fully eliminate a
434 researchers' influence on participant responses. In this case, the researcher’s gender or role as a
435 nutritionist may have influenced the discussions. Thirdly, data collection comprised FGDs with policy,
436 health management and household members in the community, excluding interviews within
437 individual households. The household level interviews would have created room to capture feeding
438 pattern, which this study did not. Future research may want to explore this and how intra-household
439 dynamics impact on the nutritional status of women. Future studies should also aim to examine the
440 correlation between gender norms and children’s nutritional status.
441 441

# 442 Acknowledgements

443 We would like to thank the participants and fieldworkers who gave their time to this study, and
444 without whom this research would not have been possible.
445 445

# 446 References

447 1.	World Health Organisation. *Malnutrition Factsheet*. World Health Organisation; 2020.
448 2.	UNICEF, WHO, World Bank Group. *Levels and Trends in Child Malnutrition: UNICEF / WHO /*
449 *World Bank Group Joint Child Malnutrition Estimates. Key Findings of the 2018 Edition*. United Nations
450 Children’s Fund (UNICEF); 2018.
451 3.	UNICEF. *Nutrition, Malnutrition: Current Status and Progress*. United Nations Children's Fund 452	(UNICEF); 2018.

453	4.	Dewey KG, Begum K. Long-term consequences of stunting in early life. *Maternal & Child*

454	*Nutrition*. 2011/10/01 2011;7(s3):5-18. doi:10.1111/j.1740-8709.2011.00349.x

455	5.	Aheto JMK, Keegan TJ, Taylor BM, Diggle PJ. Childhood Malnutrition and Its Determinants

456	among Under-Five Children in Ghana. *Paediatric	and	Perinatal	Epidemiology*. 2015/11/01 457	2015;29(6):552-561. doi:10.1111/ppe.12222

458	6.	de Onis M, Dewey KG, Borghi E, et al. The World Health Organization's global target for

459	reducing childhood stunting by 2025: rationale and proposed actions. *Matern Child Nutr*. Sep 2013;9 460	Suppl 2(Suppl 2):6-26. doi:10.1111/mcn.12075

461	7.	Bloem M, Pee Sd. Scaling up nutrition: a framework for action. *Food Nutr Bull*. 2010;31(1):178- 462	186.

463	8.	Sullivan LM, Sakayan M, Cernak K. 1,000 Days: Mobilizing Investments for Healthier, More

464	Prosperous Futures. *ZERO TO THREE*. 2018;38(4):31-36.

465	9.	Kominiarek MA, Rajan P. Nutrition Recommendations in Pregnancy and Lactation. *Med Clin*

466	*North Am*. Nov 2016;100(6):1199-1215. doi:10.1016/j.mcna.2016.06.004

467 10.	Gupta A, Suri S, Dadhich J, Trejos M, Nalubanga B. The world breastfeeding trends initiative:
468 implementation of the global strategy for infant and young child feeding in 84 countries. *Journal of*
469 *public health policy*. 2019;40(1):35-65.
470 11.	Sankar MJ, Sinha B, Chowdhury R, et al. Optimal breastfeeding practices and infant and child
471 mortality: a systematic review and meta‐analysis. *Acta paediatrica*. 2015;104:3-13.
472 12.	Cumming O, Cairncross S. Can water, sanitation and hygiene help eliminate stunting? Current
473 evidence and policy implications. *Maternal & Child Nutrition*. 2016/05/01 2016;12(S1):91-105. 474	doi:10.1111/mcn.12258

475	13.	Tickell KD, Pavlinac PB, John-Stewart GC, et al. Impact of Childhood Nutritional Status on

476	Pathogen Prevalence and Severity of Acute Diarrhea. *Am J Trop Med Hyg*. Nov 2017;97(5):1337-1344. 477	doi:10.4269/ajtmh.17-0139

478	14.	Alemayehu M, Tinsae F, Haileslassie K, Seid O, Gebregziabher G, Yebyo H. Undernutrition

479	status and associated factors in under-5 children, in Tigray, Northern Ethiopia. *Nutrition*. Jul-Aug 480	2015;31(7-8):964-70. doi:10.1016/j.nut.2015.01.013

481	15.	Reinhardt K, Fanzo J. Addressing Chronic Malnutrition through Multi-Sectoral, Sustainable

482	Approaches:	A	Review	of	the	Causes	and	Consequences.	*Front	Nutr*.	2014;1:13. 483	doi:10.3389/fnut.2014.00013

484	16.	UNICEF. UNICEF Conceptual Framework for Malnutrition. 2008.

485	17.	World Health Organisation. *UNICEF Conceptual Framework on Maternal and Child Nutrition*.

486	World Health Organisation; 2021.

487	18.	World Health Organisation. *Gender [Internet]*. <https://www.who.int/health-topics/gender>; 488	2022.

489	19.	Holmes AV, Chin NP, Kaczorowski J, Howard CR. A barrier to exclusive breastfeeding for WIC

490	enrollees: limited use of exclusive breastfeeding food package for mothers. *Breastfeeding Medicine*. 491	2009;4(1):25-30.

492	20.	Jesson J, Coulibaly A, Sylla M, et al. Evaluation of a Nutritional Support Intervention in

493	Malnourished HIV-Infected Children in Bamako, Mali. *JAIDS Journal of Acquired Immune Deficiency*

494	*Syndromes*. 2017;76:14Y 157.

495	21.	Morgan R, Tetui M, Muhumuza Kananura R, Ekirapa-Kiracho E, George AS. Gender dynamics

496	affecting maternal health and health care access and use in Uganda. *Health Policy and Planning*. 497	2017;32(suppl_5):v13-v21. doi:10.1093/heapol/czx011

498	22.	Morgan R, George A, Ssali S, Hawkins K, Molyneux S, Theobald S. How to do (or not to do)…

499	gender	analysis	in	health	systems	research. *Health	Policy	Plan*. Oct	2016;31(8):1069-78.

500	doi:10.1093/heapol/czw037

501	23.	Jhpiego. *Gender Analysis Toolkit for Health Systems*. Jhpiego; 2016.

502	24.	Warren H. Using gender-analysis frameworks: theoretical and practical reflections. *Gender &*

503	*Development*. 2007/07/01 2007;15(2):187-198. doi:10.1080/13552070701391847

504 25.	World Health Organisation. *Taking sex and gender into account in emerging infectious*
505 *diseases programmes: An analytical framework*. World Health Organisation; 2011.
506 26.	World Health Organisation. *Incorporating Intersectional Gender Analysis into Research on*
507 *Infectious Diseases of Poverty: A Toolkit for Health Researchers*. World Health Organisation; 2020.
508 27.	World Bank. World Development Indicators. 2019;
509 28.	Government of Malawi. *National Multi-Sector Nutrition Policy 2018 - 2022*. Government of
510 Malawi; 2018.
511 29.	Government of Malawi National Statistical Office. *Malawi Demographic and Health Survey*.
512 Malawi National Statistical Office; 2017.
513 30.	Committee GoMVA. *The Integrated Food Security Phase Classification (IPC) in Malawi:*
514 *Findings of the 2017 Assessment and Analysis*. Government of Malawi; 2017.
515 31.	World Food Programme. *Household Food Security in Malawi: Results from a Comprehensive*
516 *Food Security Survey* World Food Programme; 2018.
517 32.	World	Bank.	What	is	Food	Security?
518 <https://www.worldbank.org/en/topic/agriculture/brief/food-security-update/what-is-food-security>
519 33.	Dressel A, Mkandawire E, Gondwe KW, et al. The intersection of food insecurity and health
520 for rural Malawian women at the end of life. *International Journal of Palliative Nursing*. 2020/10/02 521	2020;26(7):372-382. doi:10.12968/ijpn.2020.26.7.372
522 34.	Government of Malawi, Policy AIfD. *Child Marriage in Malawi Factsheet*. Government of
523 Malawi Ministry of Gender, Children, Disability and Social Welfare; 2017.
524 35.	O’’Neil T, Kanyongolo N, Wales J, Walita Mkandawire M. *Women and Power: Representation*
525 *and Influence in Malawi’s Parliament*. Overseas Development Institute; 2016.
526 36.	Lodenstein E, Pedersen K, Botha K, Broerse JEW, Dieleman M. Gendered norms of
527 responsibility: reflections on accountability politics in maternal health care in Malawi. *International*

528	*Journal for Equity in Health*. 2018/09/24 2018;17(1):131. doi:10.1186/s12939-018-0848-3

529	37.	Stewart DW, Shamdasani PN. *Focus Groups: Theory and Practice*. SAGE; 2015.

530	38.	Hennink M, Hutter I, Bailey A. *Qualitative Research Methods*. 2nd Edition ed. SAGE

531	Publications Ltd.; 2020.

532	39.	Braun V, Clarke V. Using thematic analysis in psychology. *Qualitative Research in Psychology*. 533	2006/01/01 2006;3(2):77-101. doi:10.1191/1478088706qp063oa

534	40.	Leung L. Validity, reliability, and generalizability in qualitative research. *Journal of family*

535	*medicine and primary care*. Jul-Sep 2015;4(3):324-327. doi:10.4103/2249-4863.161306

536	41.	Kamiya Y, Nomura M, Ogino H, Yoshikawa K, Siengsounthone L, Xangsayarath P. Mothers’

537	autonomy and childhood stunting: evidence from semi-urban communities in Lao PDR. *BMC Women's*

538	*Health*. 2018/05/22 2018;18(1):70. doi:10.1186/s12905-018-0567-3

539	42.	Sibhatu KT, Qaim M. Rural food security, subsistence agriculture, and seasonality. *PloS one*. 540	2017;12(10):e0186406.

541	43.	Rankoana SA. The Use of Indigenous Knowledge in Subsistence Farming: Implications for

542	Sustainable Agricultural Production in Dikgale Community in Limpopo Province, South Africa. 2017:

543	44.	Mkandawire E, Bisai C, Dyke E, et al. A qualitative assessment of gender roles in child nutrition 544	in Central Malawi. *BMC Public Health*. 2022/07/20 2022;22(1):1392. doi:10.1186/s12889-022-13749-

545	x

546	45.	Grundy E, Kravdal Ø. Do short birth intervals have long-term implications for parental health?

547	Results from analyses of complete cohort Norwegian register data. *Journal of Epidemiology and*

548	*Community Health*. 2014;68(10):958. doi:10.1136/jech-2014-204191

549 46.	Hawkes C, Fanzo J. Nourishing the SDGs: Global nutrition report 2017. 2017;
550 47.	Nations FaAOotU. *Integrating Agriculture and Nutrition Education for Improved Young Child*
551 *Nutrition*. Food and Agriculture Organisation; 2016.
552 48.	Malawi Go. *Nutrition Handbook for Farmer Field Schools*. Ministry of Agriculture, Irrigation
553 and Water Development; 2015.
554 49.	Canavan CR, Graybill L, Fawzi W, Kinabo J. The SDGs Will Require Integrated Agriculture,
555 Nutrition,	and	Health	at	the	Community	Level.	*Food	Nutr	Bull*.	2016;37(1):112-115. 556	doi:10.1177/0379572115626617

557	50.	Mkandawire E, Hendriks SL. A qualitative analysis of men’s involvement in maternal and child

558	health as a policy intervention in rural Central Malawi. *BMC Pregnancy and Childbirth*. 2018/01/19 559	2018;18(1):37. doi:10.1186/s12884-018-1669-5

560

Figure 2	[Click here to access/download;Figure;Fig 2 Gender Norms Contributing to Stunting.docx](https://www2.cloud.editorialmanager.com/pone/download.aspx?id=36521512&guid=410d45ca-d6f9-43d5-9a85-e68f3f07d87f&scheme=1)

|  |  | **Domains of RinGS Framework for Gender Analysis** | **Corresponding Theme** | **Categories** |
| --- | --- | --- | --- | --- |
|  |  |  | Gender unequal decision making on the consumption, sale and distribution of food | Distinction of roles in IYCF |
|  |  | *Division of labour, roles and everyday practices* |  | Competing spending needs |
|  |  |  |  | Nutrition not a priority in household spending |
|  |  | *Rules and decision making* |  | Men control decisions on use of farm produce |
| **How gender**  **norms affect stunting in Ntchisi district, Malawi** |  |  |  |  |
|  |  |  |  |  |
|  |  |  |  | Mothers at health centres, fathers at home |
|  |  | *Social norms and values* | Enshrined community norms influence feeding practices underpinned by gender based violence | Household conflict and gender based violence |
|  |  |  |  | Women accept GBV: fear and avoid shame |
|  |  |  |  | Prevalence of GBV after harvesting time |
|  |  |  |  | Men’s status in the community an enduring norm |
|  |  | *Access to resources* | Policy disconnections and gaps that reinforce unequal gender norms at the structural level | Agriculture, nutrition interventions disconnected |
|  |  |  |  | Poor male involvement in nutrition interventions |
|  |  |  |  | Teen motherhood and large family sizes |
|  |  |  |  | Low education and literacy levels |

Figure 3	[Click here to access/download;Figure;Fig 3 Factors Contributing to Stunting UNICEF.docx](https://www2.cloud.editorialmanager.com/pone/download.aspx?id=36521513&guid=4e5cb904-fcbd-4282-bdb2-8e8e24868d10&scheme=1)

Figure 1	[Click here to access/download;Figure;Fig 1 UNICEF Framework of Malnutrition.docx](https://www2.cloud.editorialmanager.com/pone/download.aspx?id=36521514&guid=c755e1e7-a40c-449c-a8d8-5b79994cc376&scheme=1)

**Source**: Reinhardt K, Fanzo J. Addressing Chronic Malnutrition through Multi-Sectoral, Sustainable Approaches: A Review of the Causes and Consequences. Front Nutr. 2014;1:13.

Revised Manuscript with Track Changes

# 1 How do gender norms contribute to stunting in Ntchisi District, Malawi? A qualitative study

2 Whitney Mphangwe^1*,2^, Ann Nolan^1^, Frédérique Vallieres^2^, Mairéad Finn^3,4^
3 [mphangww@tcd.ie](mailto:mphangww@tcd.ie); [fvallier@tcd.ie](mailto:fvallier@tcd.ie); [nolana13@tcd.ie](mailto:nolana13@tcd.ie); [finnm2@tcd.ie](mailto:finnm2@tcd.ie)
4 ^1^ Trinity Centre for Global Health, School of Medicine, Trinity College Dublin
5 ^2^ Food and Nutrition Officer, Ministry of Agriculture, Irrigation and Water Development
6 ^3^ Trinity Centre for Global Health, School of Psychology, Trinity College Dublin.
7 ^4^ Department of Public Health and Epidemiology, Royal College of Surgeons in Ireland
8 *Corresponding Author 9

10

11 Whitney Mphangwe
12 Ministry of Agriculture, Irrigation and Water Development
13 Ntchisi District Agriculture Office
14 P.O. Box 37
15 Ntchisi
16 Malawi 17
18 Mairéad Finn
19 Centre for Global Health, University of Dublin, Trinity College
20 7 – 9 Leinster Street South
21 Dublin 2
22 Ireland 23

# 24 How do gender norms contribute to stunting in Ntchisi District, Malawi? A qualitative study

25 **Abstract**
26 **Background and Aim:** Despite adequate food production and nutrition intervention coverage,
27 stunting remains an enduring problem in Ntchisi, Malawi. Globally, gender and social norms are known
28 to influence nutritional outcomes in children. This study explores how gender norms contribute to
29 child stunting, in Ntchisi district, Central Malawi.
30 **Research Methods:** Informed by the UNICEF Framework for Malnutrition, nine focus group
31 discussions were conducted with a target population of mothers (n=24), fathers (n=23) purposively
32 targeted through growth monitoring sessions, and members of policy and health treatment
33 committees (n=21), spanning three different areas of Ntchisi district. Data were analysed through
34 inductive thematic analysis, guided by the framework for Research in Gender and Ethics (RinGs).
35 **Results:** Three primary themes were identified: 1) gender unequal decision making on the
36 consumption, sale and distribution of food; 2) enshrined community norms influence feeding practices
37 underpinned by gender-based violence; and 3) policy disconnections and gaps that reinforce gender
38 norms regarding nutrition. Themes encompassed practices across household, health treatment, and
39 policy level.
40 **Conclusion:** Gender norms that underpin inequalities in decision making for production and
41 consumption of food undermine children’s nourishment and contributes towards sustained child
42 malnutrition in Ntchisi. Existing policy documents should revise their guidelines to incorporate gender
43 norms as key determinants of malnutrition.
44 **Keywords:**	Stunting, Gender Norms, Malnutrition, Malawi, Children, UNICEF Conceptual
45 Framework for Malnutrition

# 46 How do gender norms contribute to stunting in Ntchisi District, Malawi? A qualitative study

47 **Introduction**
48 T~~Defined by t~~he World Health Organization (WHO) defines malnutrition as “deficiencies, excesses or
49 imbalances in a person’s intake of energy and/or nutrients”^1^ broadly ~~the concept of malnutrition~~
50 incorporating~~es~~	under-nutrition,	micro-nutrient	related	malnutrition,	and~~d~~	over-nutrition.
51 Malnutrition remains a topic of global concern, with 20% of the global under-five population (149.2
52 million) currently ‘stunted,’ that is, a low height-for-age index,^2^ and where malnutrition accounts for
53 53% of under-5 mortality.^3^ Stunting causes long-term impairment including poor school performance
54 in childhood, reduced productivity later in life, and an increased risk of chronic non-communicable
55 illnesses; and, in the worst cases, death.^4^
56 Numerous international policy commitments seek to address malnutrition. The United Nations
57 (UN) Sustainable Development Goals (SDGs), particularly SDG2, aspire to improve agriculture and food
58 systems to feed and nourish malnourished populations.^5^ Likewise, the WHO’s 2018 Global Nutrition
59 Target (GNT) sought to reduce the number of stunted children by 40%^6^ and the UN’s 2015 General
60 Assembly declared 2015-2025 to be the decade of nutrition, a commitment by member states to
61 implement policies, programs, and investments to eliminate malnutrition in all its forms. Scaling Up
62 Nutrition (SUN) – a global movement working with the UN, civil society, business and donor networks
63 to tackle malnutrition - promotes exclusive care and investment in child care globally, particularly in
64 the first 1000 days of life.^7^
65 Established determinants of malnutrition include poor maternal nutrition and young child
66 feeding practices;^8,9^ young mothers’ age at first birth, which is linked to higher parity and poorer birth
67 spacing practices;^10^ depleted nutrition for mothers experiencing successive pregnancies and
68 lactation,^8^ associated with poorer feeding practices;^11^ subclinical infections from exposures to
69 contaminated environments;^12,13^ and infectious diseases such as malaria, which can compound short
70 term loss of weight and contribute to long term stunting.^14^ Malnutrition is thus determined by the
71 complex interaction of multiple social, economic, and cultural factors. These factors are reflected in
72 UNICEF’s Conceptual Framework of Maternal and Child Nutrition (Figure 1),^15^ often considered the
73 primary global policy instrument to address the causes and consequences of malnutrition and reduce
74 its prevalence.^16^ According to this framework, *basic-level* causes operate in the broader context and
75 include socio-economic and cultural context, human capital, and access to potential resources;
76 *underlying* causes operate at household and community level, including care and feeding practices;
77 and *immediate* causes relate to direct diet intake and the presence of disease.^16^ Recently updated in
78 2020, the framework now also recognizes contributors to good nutrition as being either immediate,
79 underlying, or enabling determinants.^17^

# 80 Figure 1: UNICEF Framework for Malnutrition

81 [INSERT FIGURE ONE HERE]

82

83 Gender refers to the socially constructed roles, behaviors, activities, and attributes that a given
84 society considers appropriate for men and women.^18^ More recently, growing attention has been
85 brought to the impact of gender norms on child malnutrition and stunting.^19^ In many settings, women
86 still cannot exercise freedom and autonomy of choice, including within their own households,^14^ with
87 the extent to which a mother can make independent decisions negatively associated with the
88 likelihood of childhood stunting.^19^ Similarly, granting women greater decision-making power is
89 associated with notable improvements in their children’s height-for-age index.^20^ Likewise, analysis of
90 gender relations in norms and decision-making has recently come to the fore in explaining the
91 endurance of problematic health outcomes.^21^ Indeed, positive social and cultural norms (which
92 include gender) are recognized as one of the most enabling determinants of improved nutrition for
93 women and children.^17^
94 In recognition of the role of gender and social norms in determining nutritional outcomes in
95 children, several frameworks have been created to guide gender analyses within health and nutritional
96 programming,^22-24^ which differ in their focus on types of power or area of the health system.
97 Specifically, the Research in Gender and Ethics (RinGS) Framework articulates gender differences as
98 unfolding across four domains: access to resources; division of labour, roles, and everyday practices;
99 social norms and values; and rules and decision making.^25,26^ It is considered comprehensive for
100 examining social and structural elements of gender interacting with health.^21^

# 101 Malnutrition in Malawi

102 Categorized as one of the poorest countries in the world by the World Bank,^27^ Malawi is a Southern
103 African country home to over 18 million inhabitants. A signatory to the SUN movement since 2011,
104 the Malawian Government applies UNICEF’s Conceptual Framework for Malnutrition (1990) to
105 address malnutrition.^16^ Moreover, the Malawi Multi-Sectoral National Nutrition Policy (MSNNP) sets
106 out the country’s priority areas for implementing nutrition interventions across the country.^28^
107 Specifically, these priority areas include (i) the prevention of under-nutrition, (ii) achieving gender
108 equality, equity protection, participation and empowerment, and (iii) the treatment and control of
109 malnutrition.^28^ The MSNNP outlines best practices to end malnutrition, detailing policy and
110 implementation plans to address causes of malnutrition at household, health treatment, and policy
111 levels.^28^
112 Malawi has registered some reduction in the prevalence of stunting among children under
113 five~~While reduced~~ from 47% in 2010,^29^ to~~the prevalence of stunting in children under-five years in~~
114 ~~Malawi remains a worrying~~ 37% in 2015 (,from the most recent available data), which is still very .
115 ~~Among~~high. O~~o~~ne of the most poorly affected districts in the country is Ntchisi, where 40% of the
116 population are malnourished.^29^ Paradoxically, Ntchisi is also considered one of the most household
117 food secure districts in the country.^30,31^ Being food secure defined as people having at all times
118 physical, social and economic access to food, safely consumed in sufficient quantity and quality for
119 dietary needs and food preferences, supported by an environment of adequate water and sanitation,
120 health services and care.^32,33^ Furthermore, exclusive breastfeeding rates in Ntchisi district for infants
121 under six months (61%) exceed the global average (40%).^29,2^
122 Evidence suggests that gender norms may play a role in Ntchisi’s observed malnutrition rates.
123 For example, in 2016, 47% of women were married before 18 years and 29% of adolescent girls were
124 mothers.^29,34^ Women were also found to be five times less likely to make independent decisions when
125 compared with men, a difference linked to variation in earning power.^35^ There is evidence that
126 differences in gender norms have a detrimental impact on women’s health.^36^ *How* differences in
127 gender norms may contribute to malnourishment and stunting in children, in Ntchisi, Malawi,
128 however, remain unexplored. This study therefore aimed to explore how gender norms play a role in
129 continued stunting in Ntchisi District, Malawi, and to contribute to a revised version of the UNICEF
130 Framework for Malnutrition to better reflect gender norms.
131 131

# 132 Materials and Methods

133 *Design and Sampling*
134 We employed a qualitative design, as part of a wider examination of the causes of observed stunting
135 in Ntchisi district. We conducted nine focus group discussions (FGDs) to explore the role of gender
136 norms in persistent child stunting across three different communities. FGDs were selected as they are
137 considered valuable for generating data on group social norms, while also prioritising participants’
138 perspectives.^37^ Each community was purposely selected to achieve geographical spread. Six FGDs -
139 two in each community - were conducted with mothers and fathers as household members, one FGD
140 was conducted with participants delivering nutrition health treatment and two were conducted with
141 participants working on nutrition policy. Therefore, nine focus groups explored how gender norms
142 might impact (mal)nutrition, reflecting household, health treatment and nutrition policy spheres as
143 articulated in the Malawi MSNNP.^28^
144 In total, 68 participants were recruited, with 6-9 people represented within each FGD. The six
145 FGDs with household members comprised three with males (n=24) and three with females (n=23).
146 Females were within the reproductive age group, had at least one child under the age of five in the
147 household, and were recruited through Growth Monitoring Sessions. There was no age limitation for
148 males, who were also required to have at least one child under the age of five in the household. Men
149 were recruited from nearby social gathering venues, mostly at church choir gatherings and market
150 points via word-of-mouth by the government extension workers.
151 At health treatment level, participants (n=10) included five females and five males of the
152 Nutrition Rehabilitation Unit (NRU) Management Committee. This committee comprises nutritionist,
153 nurse, and home-help workers providing treatment to severe cases of malnutrition admitted to the
154 District Health Office. Policy level participants (n=10) also comprised five females and five males of
155 the District Agriculture Coordinating Committee (DAECC), a committee that oversees and coordinates
156 all agricultural interventions at the district level, and the District Nutrition Coordinating Committee
157 (DNCC), a committee responsible for coordinating all nutrition interventions at the district.
158 158
159 Data Collection Instruments
160 Semi-structured focus group discussion guides were developed with reference to the domains of the
161 UNICEF Framework of Malnutrition^16,17^, incorporating probes to ensure maximum investigation of
162 persistent stunting. The guides included general questions about daily activities, food and income;
163 resources and decision-making; and understanding of and perspectives on stunting. Discussions were
164 conducted by the first author in Chichewa, the local language. Two field assistants, one from the health
165 sector and the other from the agriculture sector, took notes of the participants’ answers throughout
166 the discussions as a backup for the audio recordings.
167 167
168 Data Management and Analysis
169 FGDs were recorded, transcribed verbatim,^38^ and translated from Chichewa to English with close
170 reference to audio files and notes. All personal identifiers were removed. Following a thorough review
171 of the transcripts for familiarity, analysis comprised three phases of open (i.e. inductive) coding,
172 categorization, and abstraction. Transcripts were coded according to the steps of Braun and Clarke^39^,
173 with common phrases assigned codes, then inductively organized into categories and themes. A
174 framework for gender analysis,^25,26^ informed by Research in Gender and Ethics (RinGs)^22^ guided
175 analytic insights. Interpretation of the data was enhanced by discussions between the first author,
176 the field workers, and the second author to refine analytical development and verify the conceptual
177 coherence of the codes.
178 178
179 Ethics, Validity and Reliability
180 Ethics approval was granted by the Trinity Health Policy and Management, Centre for Global Health
181 (HP&M/CGH) Research Ethics Committee (REC) (Ref.20/2029/01) and by the Ntchisi District Council
182 Secretariat (Ref 2019/01/01). Informed verbal consent was sought from participants, who were fully
183 briefed about the study aims and objectives, the voluntary nature of their participation, and that they
184 could opt out at any time without penalty.
185 The validity and reliability of the data was ensured through transcription, clear and transparent
186 coding, reflexivity, and cross-checking of themes between the lead author, the second author, and
187 two field assistants.^40^ The first author, who carried out the data collection, is a nutritionist in Ntchisi
188 District, and reflexive attention was paid to the influence of her positionality throughout the data
189 collection and analysis phases.
190 190
191 191

# 192 Results:

193 **Participant Characteristics**
194 ~~Participant mothers had a mean age of 26 while fathers had a mean age of 38~~Discussions commenced
195 with a brief introduction from all participants. From this, it was gleaned that female participants
196 ranged between 18 to 38 years of age and males ranged from 25 to 56 years of age. Participants
197 reported having five children, on average. From the introductions, it was also gleaned that m~~M~~ost
198 mothers and fathers were educated to primary level only, with some to second level. Agriculture was
199 their main source of food and income. Among committee members, half were employed by
200 government while the other half were employed by non-government organizations.

# 201 Exploring the Role of Gender Norms in Contributing to Malnutrition

202 Three core themes were identified in the data to illuminate the role of gender norms in contributing
203 to malnutrition and stunting in Ntchisi. These were: (i) gender unequal decision making on the
204 consumption, sale and distribution of food; (ii) enshrined community norms influence feeding
205 practices, underpinned by gender based violence; (iii) policy disconnections and gaps that reinforce
206 unequal gender norms regarding nutrition. Themes are presented in Figure 2, aligned to the RinGS
207 Framework for Gender Analysis.^21,22^ 208

# 209 Figure 2: Gender Norms Contributing to Stunting in Ntchisi District, Malawi

210 [INSERT FIGURE TWO HERE]

# 211 Gender Unequal Decision Making Shapes the Household Production and Consumption of Food

212 Power differences between fathers and mothers affected decisions about how farm produce is
213 utilized. The policy level focus groups explained how practices in subsistence agriculture affect what
214 to produce, the quantity to produce, and where to sell produce, all of which affect how much remains
215 for families to eat. Women in the district were reported to have lower decision-making power about
216 food production by comparison to men:
217 “Men dominate…they control what is cultivated and what to do with the produce. You find that
218 a woman might know that legumes are nutritious and essential for child growth… [but] when
219 the husband wants to sell, the [wife] will not resist.” DAECC FGD.
220 Describing their social context, household participants told of the different roles that mothers and
221 fathers take and how this impacts children’s nutrition. The participants recounted that within the
222 household, fathers are considered providers while mothers are nurturers. When describing their daily
223 routine, mothers mentioned household chores like cooking, feeding, laundry, tidying the house, and
224 collecting firewood and water, the latter usually from a great distance. On the other hand, fathers
225 reported daily work of gardening, casual tasks, socializing, and sourcing income for the household.
226 Fathers and mothers emphasised a clear distinction of roles in feeding children:
227 “Men search for food…we stay at home taking care of the children… cooking for and feeding
228 them. When men bring food, we cook.” Mothers FGD
229 “We take care of children in the sense that we search for food so that woman can cook it for
230 the children.” Fathers FGD.
231 However, fathers were characterized by participants as not health or nutrition minded in relation to
232 expenditure. Almost all mothers’ groups described that men in the community use their income on
233 pleasures rather than on actual needs.
234 “Sometimes, you can work hard to produce more. However, when you harvest and sell, the man
235 will spend it on drinking and other women. You cannot question how they spend it. They forget
236 about their own children. You face it all because the children usually stay at home with a
237 mother”. Mothers FGD.
238 Similarly, fathers indicated that when they harvest, they have several responsibilities to address.
239 Nutrition might not usually be the priority.
240 “A poor person always has a handful of things to take care of. When you get money…you don’t
241 remember what nutrition or nutritious food is about” Fathers FGD.
242 Differences between mothers and fathers in decision making about utilization of farm produce also
243 manifested at the health center. The health committee FGD recounted how in-patient cases of
244 malnutrition at the health centre are presented mostly by mothers, who attend with their children
245 while fathers stay at home. Extended stays can cause worry to mothers about further disruption to
246 home life and the oversale of food:
247 “The mothers wait for children here…we give them health talks every morning. Fathers usually
248 stay at home…[but]…when the mothers stay for some days, they start complaining thinking that
249 their husbands will marry another woman or get a chance to sell out farm produce and other
250 household assets. They just become so eager to go, and it is evident in how they act.” NRU FGD.
251 Although mothers in Ntchisi were primarily responsible for feeding children, they lacked decision-
252 making power over the production and consumption of food. Fathers, who held decision making
253 power, were not oriented towards nutritional needs for children. Furthermore, inequality in decision
254 making about food for children intensifies when women are not present.
255 255

# 256 Enshrined Community Norms Influence Feeding Practices, Underpinned by Gender Based Violence

257 Household focus groups reported that inequity in household decision making around food was
258 preserved by traditional norms of gender-based violence (GBV) in Ntchisi district. A clear and strong
259 description by participants was that women cooperate with men’s decisions for fear of GBV, and do
260 not speak out about GBV in fear of shaming their husbands and losing respect.
261 “In this area, when a woman has a brilliant idea, men do not want to appear cheap, so they do
262 not agree, whatever it is about…as a woman you just coil up and keep quiet otherwise you will
263 be beaten” Mothers FGDs
264 The supremacy of this social norm was bolstered by the status it confers on men in the community.
265 “Men here say things, and that is final…maybe it could be one out of ten men that would be
266 under petticoat government. If you allow your wife to be key in making decisions, your friends
267 will despise you…even young children would look down on you.” Fathers FGD.
268 District committee members recounted that cases of GBV are more prevalent at times of the year
269 when produce is sold. Gaps in power between mothers and fathers extended to a firm inequality in
270 autonomy, which impacted on children’s nutrition.
271 “Men are just bad. During the cultivating period, they work well with their wives, but when it is
272 selling time, they prefer to work alone and spend the money on drinking or other women…this
273 initiates conflicts that usually ends in abuse. Children are usually left with mothers that do not
274 have food supplies or money.” DNCC FGD.
275 Mothers and fathers highlighted that the clusters of friends and neighbours they lived among were
276 their primary sources of knowledge, indicating the strong traditional norms shaped how children were
277 taken care of. Gender based violent practices were encased in traditional community norms.
278 “Everybody does that…that’s how we do things here. Our parents teach us what they did so that
279 we grew up. When your child is born, they take you aside and teach you many things	we
280 apply that so that our children grow up too” Fathers FGD.
281 Thus, community adherence to traditional practice creates a challenge to do things differently
282 when feeding children. Rigid social norms of women required to be deferential to men were held in
283 place by traditional expectations and the threat of gender-based violence. These norms retained an
284 enduring social power in the community and impacted practices and decision making around food for
285 children.
286 286

# 287 Policy Disconnections and Gaps that Structurally Reinforce Gender Norms Regarding Nutrition

288 A disconnection between production and nutrition interventions was articulated by policy level
289 participants, with agricultural produce sold by subsistence farmers rather than retained for their
290 families. Participants explained that while the agriculture committee incorporates nutrition
291 interventions to its activities in theory; no nutrition achievements could be attributed to this work.
292 “We try to incorporate nutrition in our planning, but all detailed plans and achievements are
293 with the DNCC because we mainly focus on agriculture … however, nutrition is for DNCC, so
294 consult them for specific achievements.” DAECC FGD.
295 The disconnection indicates a wider aspect of the gender gap because agriculture interventions -
296 usually organized by men in the district - are implemented separately from general nutrition
297 interventions, targeting women as primary caregivers for children.
298 Men’s participation in the implementation of nutrition interventions was noted as a gap by
299 household level participants. Mothers encouraged targeting fathers for inclusion, highlighting that if
300 men were aware of nutrition issues, they would be more supportive at home and more informed
301 about nutrition-related decisions.
302 “Men should be taught. They are stubborn and irresponsible because they do not know and
303 understand. They should hear for themselves. They should be included in care groups. Now care
304 groups are only full of women” Mothers FGD
305 Despite father’s lack of involvement in nutrition reflecting the socio-cultural norms in Ntchisi,
306 men in the fathers’ focus groups indicated their willingness to participate in the sessions and learn
307 more about nutrition.
308 “The extension workers always teach women. The next training, please include us…we will come
309 and are happy to learn.” Fathers FGD
310 Impacts of trainings were also affected by education levels, specifically literacy, of the
311 population. Low levels of education were reported as a general feature of the population in Ntchisi,
312 affecting practices towards childcare. Health treatment level participants highlighted how literacy
313 levels affect the uptake of trainings.
314 “You can have a hospital, offer training in all these things…however, without school, it is all in
315 vain. You need some [education] to understand the importance of it all.” NRU FGD.
316 The Government’s role was further emphasized in reducing teen pregnancies and achievements
317 in nutrition education. Teen mothers face challenges in adequately nourishing themselves and their
318 children. Pressure is exacerbated when women have their first child at a young age, because it leads
319 to larger family size. Care demands of young children cannot be met if the mother has many children,
320 closely spaced together.
321 “I think the issue is that people have many children. You see some have eight, others nine. They
322 cannot afford to monitor if all ate or not. You would not know that one missed food because
323 they are too many..[…]… the Government should do more in reducing teen pregnancies…and
324 should invest in keeping girls in school.” NRU FGD.
325 Low levels of education undermine the household’s ability to meet the nutrition needs of children,
326 affecting both choices in health and well-being and the priority given to well-being. These trends are
327 also an indication of a broader structural context with unequal gender norms impacting women and
328 children.

# 329 Discussion ~~and Conclusion~~

330 Despite a context of food security, interventions on nutritious feeding, and the development of
331 policies to target the eradication of undernutrition, children’s inadequate consumption of food in
332 Ntchisi is impacted by gendered practices and power dynamics. Specifically, differing roles between
333 men and women shape use of farm produce and feeding practices, enduring traditional social norms
334 preserved via the threat of gender-based violence and structurally bolstered through policy
335 disconnections, are all experienced as gender-related factors that impact on the nutrition of children
336 in Ntchisi.
337 Similar to Alemayehu et al.^14^, and Kamiya et al.^41^, women interviewed in Ntchisi district also had
338 limited power in making decisions about use of food within their households. Adhering to men’s
339 decisions regarding food production is a strong tradition that results in households being unable to
340 consume the right varieties and quantities. Although subsistence farming is generally characterized by
341 the consumption of produce,^42^ the narratives in this study highlighted how food produced for
342 subsistence is sold and distributed rather than consumed.	Subsistence farming also connotes
343 adherence to indigenous knowledge and traditions in production and consumption, pointing again to
344 the embedded nature of traditional practices.^43^
345 Power differences between women and men were also evident in that women were involved
346 in harvesting food, but excluded from decisions on the use of the harvest. Conflict related to decisions
347 on use of harvest produce reportedly resulted in increased gender-based violence or a rupture in the
348 marriage, as participants recounted risks of both increased violence and fathers sometimes
349 abandoning their families. This trend has been identified elsewhere, where women characterized
350 themselves as ‘harvest wives’ in cases where their husbands abandoned them when the harvest was
351 complete.^44^ A salient phenomenon that impacts the use of food is women’s lack of inclusion in
352 decisions on how harvested food will be used for the family, with a heightened risk of gender based
353 violence or relationship breakdown at this point.
354 While the government has an established role in imparting knowledge on food preparation and
355 feeding, enshrined gender norms undermine how women can practice their learning, as they are
356 pressured to adhere to men’s decisions. Women’s translation of their nutrition knowledge into
357 practice is also challenged by the high number of GBV cases reported in the district, reported as more
358 prevalent during harvest time, also contributing to the limited contribution of agricultural produce to
359 nourishment of children. Consistent with previous findings, children’s dietary intake in Ntchisi is also
360 reportedly further compromised by mothers’ young age and a large number of children, with
361 consequent reduced care practices.^45^ In the context where women do not make independent
362 decisions, it can be assessed that the lack of independence extends to decisions on family size and
363 birth control methods.
364 Father’s lack of involvement in their child’s nutrition within Ntchisi was also found to be a
365 manifestation of the social cultural norms in the district. Participants across the sample suggested
366 inclusion of men in community trainings on infant and young child feeding. In a social context where
367 fathers want to be respected for not being “under petticoat government”, the cultural change
368 required to incorporate men into nutrition interventions is evident. A more targeted approach to
369 addressing men’s roles in nutrition, that takes account of the bearing of gender norms, is required.
370 In support of the UNICEF Conceptual Framework of Malnutrition, the findings from this study
371 demonstrate that gender dynamics indeed shape malnutrition across all three causes of malnutrition
372 articulated in the framework - basic, underlying, and intermediate. Practices at the underlying levels
373 of household and community related to the production and consumption of food were highly unequal
374 for men and women. At the basic level of socio-economic and cultural context, capital, and access to
375 resources, powerful norms bolstering inequity between women and men were evident. Unequal
376 practices within these domains were highly gendered, intersecting to shape lack of access to and use
377 of resources for the nourishment of children. These findings and considerations are presented in
378 Figure Three as an expanded version of the UNICEF Conceptual Framework for Maternal and Child
379 Malnutrition.

# 380 Figure 3: Factors Contributing to Stunting adapted to the 1990 UNICEF Conceptual Framework of

381 **Malnutrition**
382 [INSERT FIGURE 3 HERE]
383 The current study adds to the existing literature by illustrating that in Ntchisi, gender norms
384 manifest across basic, underlying, and intermediate levels, and not just at the basic level as currently
385 articulated within the UNICEF conceptualization. Accordingly, gender should be articulated across the
386 entire UNICEF	framework, and	the	framework	might	benefit	from	applying	the	WHO’s
387 recommendations for incorporating intersectional gender analysis.^25,26^ In line with the domains within
388 RiNGs framework, domains of ‘access to resources’; ‘divisions of labour, roles and everyday practices’;
389 ‘social norms and values’; ‘rules and decision making’^26^ should also be incorporated into the UNICEF
390 framework to illuminate how unequal gender norms and roles unfold to explain continued problems
391 of nutrition for children.
392 The lack of policy coordination between agriculture and nutrition could contribute to
393 explanations of the disconnection between food production, food consumption, and nutrition status
394 in Ntchisi. Strengthening policy linkages between these domains would align with the 2017 Global
395 Nutrition Report^46^ statement that achieving good nutrition means practicing agriculture that feeds
396 and nourishes a nation state. The Food and Agriculture Organization emphasizes the importance of
397 integrating food production and nutrition interventions in parallel, and the findings presented here
398 provide insights for practical implementation.^47,48^ Integrating nutrition and agriculture is emphasized
399 by the theme of the 2016 World Food Day (WFD) commemorations; “our health is not only what we
400 eat, it is primarily what we grow”.^49^ Gender sensitivity must traverse these connections. Male
401 involvement in these connections is imperative, in recognition of the fact that they hold decision-
402 making power and asset ownership. Mkandawire & Hendricks (2015) note that while it is important
403 to target women and children in nutrition interventions, the exclusion of men ignores the fact that
404 women are limited in terms of decision-making powers and asset ownership.^50^ 405
406 ~~Taken together, our findings demonstrate how (mal)nutrition is impacted by the socially~~
407 ~~constructed roles, behaviours, activities and attributes that a given society considers appropriate for~~
408 ~~men and women, which can manifest as unequal power relations shaping access to and use of food.~~^26^
409 The Malawi MSNNP cites gender equality, equity protection, participation and empowerment^28^ as its
410 second priority area. The findings can be translated into policy actions on under-nutrition and stunting
411 under the umbrella of the MSNNP. Specifically, government and development partners could continue
412 multi-sectoral implementation but place special focus on male involvement.
413 The Malawi MSNNP 2018-2022 emphasizes the need to involve all line ministries and
414 stakeholders in reducing child under-nutrition. In line with the policy objectives, this study
415 recommends that Government and other NGOs connect programmes across household, health
416 treatment and policy levels in Ntchisi; connect agriculture and nutrition polices; and incorporate a
417 gender lens. In addition, education must underpin all interventions, especially so for girls experiencing
418 teen pregnancy. The successful implementation of nutrition programmes relies on an educated and
419 literate population.
420 Conclusion
421 In conclusion, the findings of this study have demonstrated how (mal)nutrition is impacted by the
422 socially constructed roles, behaviours, activities and attributes that a given society considers
423 appropriate for men and women, which can manifest as unequal power relations shaping access to
424 and use of food.^26^ The unequal division of power and resources between men and women in Ntchisi
425 district in the central region of Malawi is a contributing factor to levels of malnutrition and stunting.
426 To address these influences on (mal)nutrition, global and national policy documents can integrate
427 gender sensitive approaches cutting across other policy areas, allowing gender to be fully integrated
428 in response. it appears appropriate to recommend that intersectional gender analysis that takes
429 account of gender inequality at household and community levels, should be mainstreamed into the
430 UNICEF framework as also recommended by the WHO. Efforts to improve child nutrition need to be
431 aligned with gender interventions to improve access, utilization and consumption of nutritious foods.
432 UNICEF’s conceptual	framework	of	malnutrition	should	highlight	gender	for	more	holistic
433 programming to reflect that improving the nutrition status of children in a highly productive
434 agricultural region is only possible if gender and nutrition interventions are indissolubly linked.

# 435 Limitations

436 The research had several limitations. Firstly, interviews were conducted in Chichewa, the vernacular
437 language in Ntchisi, and transcripts were translated to English by the first author who speaks Chichewa
438 and English fluently. This translation process, however, might have introduced some mis-explanations
439 of meanings of terms. Second, the first author is a nutrition officer who works in the area under study.
440 While every effort was made to account for the researcher role, it is challenging to fully eliminate a
441 researchers' influence on participant responses. In this case, the researcher’s gender or role as a
442 nutritionist may have influenced the discussions. Thirdly, data collection comprised FGDs with policy,
443 health management and household members in the community, excluding interviews within
444 individual households. The household level interviews would have created room to capture feeding
445 pattern, which this study did not. Future research may want to explore this and how intra-household
446 dynamics impact on the nutritional status of women. Future studies should also aim to examine the
447 correlation between gender norms and children’s nutritional status.
448 448

# 449 Acknowledgements

450 We would like to thank the participants and fieldworkers who gave their time to this study, and
451 without whom this research would not have been possible.
452 452

# 453 References

454 1.	World Health Organisation. *Malnutrition Factsheet*. World Health Organisation; 2020.
455 2.	UNICEF, WHO, World Bank Group. *Levels and Trends in Child Malnutrition: UNICEF / WHO /*
456 *World Bank Group Joint Child Malnutrition Estimates. Key Findings of the 2018 Edition*. United Nations
457 Children’s Fund (UNICEF); 2018.
458 3.	UNICEF. *Nutrition, Malnutrition: Current Status and Progress*. United Nations Children's Fund 459	(UNICEF); 2018.

460	4.	Dewey KG, Begum K. Long-term consequences of stunting in early life. *Maternal & Child*

461	*Nutrition*. 2011/10/01 2011;7(s3):5-18. doi:10.1111/j.1740-8709.2011.00349.x

462	5.	Aheto JMK, Keegan TJ, Taylor BM, Diggle PJ. Childhood Malnutrition and Its Determinants

463	among Under-Five Children in Ghana. *Paediatric and	Perinatal	Epidemiology*. 2015/11/01 464	2015;29(6):552-561. doi:10.1111/ppe.12222

465	6.	de Onis M, Dewey KG, Borghi E, et al. The World Health Organization's global target for

466	reducing childhood stunting by 2025: rationale and proposed actions. *Matern Child Nutr*. Sep 2013;9 467	Suppl 2(Suppl 2):6-26. doi:10.1111/mcn.12075

468	7.	Bloem M, Pee Sd. Scaling up nutrition: a framework for action. *Food Nutr Bull*. 2010;31(1):178- 469	186.

470	8.	Sullivan LM, Sakayan M, Cernak K. 1,000 Days: Mobilizing Investments for Healthier, More

471	Prosperous Futures. *ZERO TO THREE*. 2018;38(4):31-36.

472	9.	Kominiarek MA, Rajan P. Nutrition Recommendations in Pregnancy and Lactation. *Med Clin*

473	*North Am*. Nov 2016;100(6):1199-1215. doi:10.1016/j.mcna.2016.06.004

474 10.	Gupta A, Suri S, Dadhich J, Trejos M, Nalubanga B. The world breastfeeding trends initiative:
475 implementation of the global strategy for infant and young child feeding in 84 countries. *Journal of*
476 *public health policy*. 2019;40(1):35-65.
477 11.	Sankar MJ, Sinha B, Chowdhury R, et al. Optimal breastfeeding practices and infant and child
478 mortality: a systematic review and meta‐analysis. *Acta paediatrica*. 2015;104:3-13.
479 12.	Cumming O, Cairncross S. Can water, sanitation and hygiene help eliminate stunting? Current
480 evidence and policy implications. *Maternal & Child Nutrition*. 2016/05/01 2016;12(S1):91-105. 481	doi:10.1111/mcn.12258

482	13.	Tickell KD, Pavlinac PB, John-Stewart GC, et al. Impact of Childhood Nutritional Status on

483	Pathogen Prevalence and Severity of Acute Diarrhea. *Am J Trop Med Hyg*. Nov 2017;97(5):1337-1344. 484	doi:10.4269/ajtmh.17-0139

485	14.	Alemayehu M, Tinsae F, Haileslassie K, Seid O, Gebregziabher G, Yebyo H. Undernutrition

486	status and associated factors in under-5 children, in Tigray, Northern Ethiopia. *Nutrition*. Jul-Aug 487	2015;31(7-8):964-70. doi:10.1016/j.nut.2015.01.013

488	15.	Reinhardt K, Fanzo J. Addressing Chronic Malnutrition through Multi-Sectoral, Sustainable

489	Approaches:	A	Review	of	the	Causes	and	Consequences.	*Front	Nutr*.	2014;1:13. 490	doi:10.3389/fnut.2014.00013

491	16.	UNICEF. UNICEF Conceptual Framework for Malnutrition. 2008.

492	17.	World Health Organisation. *UNICEF Conceptual Framework on Maternal and Child Nutrition*.

493	World Health Organisation; 2021.

494	18.	World Health Organisation. *Gender [Internet]*. <https://www.who.int/health-topics/gender>; 495	2022.

496	19.	Holmes AV, Chin NP, Kaczorowski J, Howard CR. A barrier to exclusive breastfeeding for WIC

497	enrollees: limited use of exclusive breastfeeding food package for mothers. *Breastfeeding Medicine*. 498	2009;4(1):25-30.

499	20.	Jesson J, Coulibaly A, Sylla M, et al. Evaluation of a Nutritional Support Intervention in

500	Malnourished HIV-Infected Children in Bamako, Mali. *JAIDS Journal of Acquired Immune Deficiency*

501	*Syndromes*. 2017;76:14Y 157.

502	21.	Morgan R, Tetui M, Muhumuza Kananura R, Ekirapa-Kiracho E, George AS. Gender dynamics

503	affecting maternal health and health care access and use in Uganda. *Health Policy and Planning*. 504	2017;32(suppl_5):v13-v21. doi:10.1093/heapol/czx011

505	22.	Morgan R, George A, Ssali S, Hawkins K, Molyneux S, Theobald S. How to do (or not to do)…

506	gender	analysis	in	health	systems	research. *Health	Policy	Plan*. Oct	2016;31(8):1069-78.

507	doi:10.1093/heapol/czw037

508	23.	Jhpiego. *Gender Analysis Toolkit for Health Systems*. Jhpiego; 2016.

509	24.	Warren H. Using gender-analysis frameworks: theoretical and practical reflections. *Gender &*

510	*Development*. 2007/07/01 2007;15(2):187-198. doi:10.1080/13552070701391847

511 25.	World Health Organisation. *Taking sex and gender into account in emerging infectious*
512 *diseases programmes: An analytical framework*. World Health Organisation; 2011.
513 26.	World Health Organisation. *Incorporating Intersectional Gender Analysis into Research on*
514 *Infectious Diseases of Poverty: A Toolkit for Health Researchers*. World Health Organisation; 2020.
515 27.	World Bank. World Development Indicators. 2019;
516 28.	Government of Malawi. *National Multi-Sector Nutrition Policy 2018 - 2022*. Government of
517 Malawi; 2018.
518 29.	Government of Malawi National Statistical Office. *Malawi Demographic and Health Survey*.
519 Malawi National Statistical Office; 2017.
520 30.	Committee GoMVA. *The Integrated Food Security Phase Classification (IPC) in Malawi:*
521 *Findings of the 2017 Assessment and Analysis*. Government of Malawi; 2017.
522 31.	World Food Programme. *Household Food Security in Malawi: Results from a Comprehensive*
523 *Food Security Survey* World Food Programme; 2018.
524 32.	World	Bank.	What	is	Food	Security?
525 <https://www.worldbank.org/en/topic/agriculture/brief/food-security-update/what-is-food-security>
526 33.	Dressel A, Mkandawire E, Gondwe KW, et al. The intersection of food insecurity and health
527 for rural Malawian women at the end of life. *International Journal of Palliative Nursing*. 2020/10/02 528	2020;26(7):372-382. doi:10.12968/ijpn.2020.26.7.372
529 34.	Government of Malawi, Policy AIfD. *Child Marriage in Malawi Factsheet*. Government of
530 Malawi Ministry of Gender, Children, Disability and Social Welfare; 2017.
531 35.	O’’Neil T, Kanyongolo N, Wales J, Walita Mkandawire M. *Women and Power: Representation*
532 *and Influence in Malawi’s Parliament*. Overseas Development Institute; 2016.
533 36.	Lodenstein E, Pedersen K, Botha K, Broerse JEW, Dieleman M. Gendered norms of
534 responsibility: reflections on accountability politics in maternal health care in Malawi. *International*

535	*Journal for Equity in Health*. 2018/09/24 2018;17(1):131. doi:10.1186/s12939-018-0848-3

536	37.	Stewart DW, Shamdasani PN. *Focus Groups: Theory and Practice*. SAGE; 2015.

537	38.	Hennink M, Hutter I, Bailey A. *Qualitative Research Methods*. 2nd Edition ed. SAGE

538	Publications Ltd.; 2020.

539	39.	Braun V, Clarke V. Using thematic analysis in psychology. *Qualitative Research in Psychology*. 540	2006/01/01 2006;3(2):77-101. doi:10.1191/1478088706qp063oa

541	40.	Leung L. Validity, reliability, and generalizability in qualitative research. *Journal of family*

542	*medicine and primary care*. Jul-Sep 2015;4(3):324-327. doi:10.4103/2249-4863.161306

543	41.	Kamiya Y, Nomura M, Ogino H, Yoshikawa K, Siengsounthone L, Xangsayarath P. Mothers’

544	autonomy and childhood stunting: evidence from semi-urban communities in Lao PDR. *BMC Women's*

545	*Health*. 2018/05/22 2018;18(1):70. doi:10.1186/s12905-018-0567-3

546	42.	Sibhatu KT, Qaim M. Rural food security, subsistence agriculture, and seasonality. *PloS one*. 547	2017;12(10):e0186406.

548	43.	Rankoana SA. The Use of Indigenous Knowledge in Subsistence Farming: Implications for

549	Sustainable Agricultural Production in Dikgale Community in Limpopo Province, South Africa. 2017:

550	44.	Mkandawire E, Bisai C, Dyke E, et al. A qualitative assessment of gender roles in child nutrition 551	in Central Malawi. *BMC Public Health*. 2022/07/20 2022;22(1):1392. doi:10.1186/s12889-022-13749-

552	x

553	45.	Grundy E, Kravdal Ø. Do short birth intervals have long-term implications for parental health?

554	Results from analyses of complete cohort Norwegian register data. *Journal of Epidemiology and*

555	*Community Health*. 2014;68(10):958. doi:10.1136/jech-2014-204191

556 46.	Hawkes C, Fanzo J. Nourishing the SDGs: Global nutrition report 2017. 2017;
557 47.	Nations FaAOotU. *Integrating Agriculture and Nutrition Education for Improved Young Child*
558 *Nutrition*. Food and Agriculture Organisation; 2016.
559 48.	Malawi Go. *Nutrition Handbook for Farmer Field Schools*. Ministry of Agriculture, Irrigation
560 and Water Development; 2015.
561 49.	Canavan CR, Graybill L, Fawzi W, Kinabo J. The SDGs Will Require Integrated Agriculture,
562 Nutrition,	and	Health	at	the	Community	Level.	*Food	Nutr	Bull*.	2016;37(1):112-115. 563	doi:10.1177/0379572115626617

564	50.	Mkandawire E, Hendriks SL. A qualitative analysis of men’s involvement in maternal and child

565	health as a policy intervention in rural Central Malawi. *BMC Pregnancy and Childbirth*. 2018/01/19 566	2018;18(1):37. doi:10.1186/s12884-018-1669-5

567

Response to Reviewers

| **REVIEWERS COMMENTS** | **REQUIRED ACTION** | **AUTHOR’S RESPONSE** |
| --- | --- | --- |
| **R1** |  |  |
| Congratulations to the authors. The paper is an interesting read and is well written in a scientific manner. The paper addresses an important subject matter of malnutrition and gender roles are indeed a mater issues that needs  to be addressed |  | Thank you for your time and consideration in reviewing this piece. We appreciate your feedback and believe that the manuscript is greatly improved as a result of the reviewer comments. Thank you again for your time in reviewing this. |
| **R2** |  |  |
| In the abstract, the study appears not to have target population. |  | Thank you for this feedback. At community level, the target population was fathers and mothers at household level. At district/implementation level, the target population were the coordinating committees for nutrition and agriculture. At health management level, the target population was the NRU for health management. These have now been summarized and reported in the  abstract under research methods. |
| Lines 48& 49 Defined by the World Health Organization (WHO) as  “deficiencies, excesses or  imbalances in a person’s intake of energy and/or nutrients | ”- What was defined by WHO? The statement needs to be reconstructed. | Thank you for this observation, we have amended this. |
| Line 112 While reduced from 47% in 2010,(29) the prevalence of stunting in children under-five  years in……………….. | This phrase might not be appropriate to start a paragraph, please  kindly re-phrase | Thank you for your observation and feedback. This statement has been rephrased. It is now on line 116. |
| Line 113. Among one of the most poorly………………… | You might need to delete among | Thank you for this observation, we  have amended this. This phrase is now on line 119. |
| Lines 181-183 Ethics approval was granted by the Trinity Health Policy and Management, Centre for Global Health (HP&M/CGH) Research Ethics Committee (REC) and by the Ntchisi District Council Secretariat, including the District Health Office (DHO) and the  District Agriculture Office (DAO) | Insert the study’s ethical approval number | Reference Numbers have been added for both RECs. |
| Informed verbal…………… |  | Thank you for this observation, we  have amended this. |

| Line 199 a mean age of 26 while fathers had a mean age of 38.  Participants reported………… | Mean must be reported with its SD value | This information was gleaned from the focus group discussions rather than calculated based on the use of a demographic questionnaire. We have  amended the language to reflect this in the manuscript. |
| --- | --- | --- |
| Lines 200 & 201 Most mothers and fathers were educated to primary level only, with some to second level. | What’s the percentage figure?  Percentage should also be reported to quantify the difference | Thank you. This information was also gleaned from the brief introductions by the participants at the outset of the focus group discussions. The manuscript has been amended to reflect this on line 202. |
| Line 332 Discussion and Conclusion | Conclusion must be separate as contained in the author’s guide for this journal | Thank you for noting this. This has been amended. |
| Kindly insert the DOIs for the cited articles, and effect all necessary corrections by providing the missing information in the references |  | Thank you for this feedback. This has been amended as reflected in the references section. The Vancouver style as stipulated by PLOS ONE does not include DOIs. We have adjusted to another numeric style, JAMA, which  does include the DOIs. |
| The topic and the discuss appears not to tally, there are no information on feeding pattern of the children and their nutritional status. |  | Thank you for this feedback. The discussion focused on the three main themes in the findings; decision- making around production and consumption, norms around feeding practices and the policy gaps fostering gender norms as fully presented in the results section. The research did not focus discussion around feeding patterns as children were accessed through growth monitoring sessions. These sessions screen children for malnutrition; on which data was already presented demonstrating that the levels of malnutrition are generally high in this area. The absence of information on feeding patterns has been added as a limitation in this study and a recommendation for future studies that research is conducted at household level to  capture feeding patterns. |

| Secondly, is the study a purposive sampling of parents with stunted children? |  | Thank you for this observation. This study purposively targeted mothers and fathers at growth monitoring sessions in an area where children were generally stunted, as well as coordinating/management level committees. The first author was a district nutritionist and was familiar with the general experience of under-  nutrition as captured through the growth monitoring sessions. |
| --- | --- | --- |
| Thirdly, there is no correlational study to show the impact of gender norm on children’s nutritional status, so the study appears to be inconclusive. |  | We appreciate this feedback and the time you took to review this document.  This study was a qualitative investigation of the way in which gender roles shape access to resources and impact child stunting. Rather than defining and measuring impact, qualitative studies seek to highlight the processes by which particular phenomena occur and the meaning ascribed to that phenomena by participants.  We have added the lack of correlation as a limitation and recommended that future studies should seek to examine  this. |
